# Disordered descent into sleep: microstructural divergence across arousal-linked conditions

**DOI:** 10.1038/s44323-026-00070-8

**Published:** 2026-02-26

**Authors:** Nazanin Biabani, Adam Birdseye, Valentina Gnoni, Riva Tauman, Katarina Ilic, Panagis Drakatos, Alexander Nesbitt, Monica Puligheddu, David O’Regan, Robert Leech, Peter J. Goadsby, Ivana Rosenzweig

**Affiliations:** 1 Sleep and Brain Plasticity Centre, Department of Neuroimaging, Institute of Psychiatry, Psychology and Neuroscience (IoPPN), King’s College London, London, UK; 2 Sleep Disorders Centre, NHS Guy’s and St Thomas’ Hospital, London, UK; 3 Department of Clinical Research in Neurology, Center for Neurodegenerative Diseases and the Aging Brain, Pia Fondazione “Card. G. Panico”, Tricase, LE Italy; 4 Sieratzki-Sagol Institute for Sleep Medicine, Tel Aviv Sourasky Medical Center, Tel Aviv, Israel; 5 Department of Neuroimaging, BRAIN Centre, Institute of Psychiatry Psychology and Neuroscience, King’s College London, London, UK; 6 School of Basic and Medical Biosciences, Faculty of Life Science and Medicine, King’s College London, London, UK; 7 Department of Neurology, Guy’s and St Thomas’ NHS Foundation Trust, London, UK; 8 Sleep and Epilepsy Centre, Department of Medical Sciences and Public Health University of Cagliari, Cagliari, Italy; 9 Centre for Neuroimaging Science, King’s College London, London, UK; 10 NIHR-Wellcome Trust King’s Clinical Research Facility, King’s College London, London, UK; 11 Division of Biomedical Sciences, King Abdullah University of Science and Technology, Thuwal, Saudi Arabia

**Keywords:** Neurology, Neuroscience

## Abstract

Sleep onset is an unstable transition whose microstructure may distinguish clinical phenotypes. Using Hori 4-second microstaging in patients with narcolepsy type 1, idiopathic REM sleep behaviour disorder, NREM parasomnia and an exploratory fibromyalgia cohort, each with matched controls (*n* = 48 pairs), we quantified timing, entropy, hemispheric laterality and sequence ordering. Narcolepsy showed compressed, irregular onset, fibromyalgia prolonged divergent onset, and the remaining groups near-normative trajectories, suggesting disorder-specific signatures of arousal instability.

The transition from wakefulness to sleep is not merely a passive drift, but a dynamic reorganisation of brain state involving rapid changes in cortical synchrony, thalamo-cortical coupling, neuromodulatory tone, and behavioural responsiveness^1–4^. Rather than following a uniform or linear trajectory, this transition is marked by brief reversals, local instabilities, and heterogeneous intermediate states that can vary widely between individuals and across nights^5–8^. Characterising this fine-grained “descent into sleep” has become an important goal in both basic sleep physiology and clinical neuroscience^9,10^.

Traditional sleep staging, as defined by the American Academy of Sleep Medicine (AASM), segments the night into 30-s epochs assigned to wake, N1, N2, N3 or REM^11,12^. This framework has transformed clinical practice by providing a standardised language for sleep architecture, but its coarse temporal resolution obscures transient features that unfold over a few seconds, such as micro-arousals, local slow waves, alpha–theta shifts, and brief REM intrusions. It may therefore underestimate the complexity and instability of the wake–sleep transition, particularly in populations with atypical neurophysiology^13,14^.

To address these limitations, the Hori staging system offers a 10-stage microstructural description of sleep onset based on 4-s EEG epochs^15–18^. It tracks the evolution from relaxed wakefulness with dominant alpha, through mixed alpha–theta and early theta-dominant periods (stages 4–8), to the appearance of vertex waves and K-complex-like elements characteristic of N1/N2 (stages 9–10). Hori staging has been used to study narcolepsy, insomnia, hypnotic effects, and subtle changes in vigilance and drowsiness^19–22^, but systematic characterisation across diagnostic populations remains limited and cross-disorder comparisons are rare^9^.

In parallel, recent conceptual models have begun to frame sleep onset as a nonlinear state transition rather than a gradual slide^5,21–25^. Within this view, wake, N1 and N2 can be thought of as quasi-stable attractor-like states in a low-dimensional state space, and the wake–sleep transition as a passage through a noisy, metastable boundary region. Work on local sleep, arousal instability, and critical transitions suggests that small perturbations in neuromodulatory tone or network connectivity may shift the system between alternative trajectories, with consequences for subjective awareness and responsiveness^20,26–29^. These ideas have clear implications for neuropsychiatry, but remain underexplored in the context of clinical neurophysiology.

Clinically, the sleep onset process is frequently altered in conditions such as narcolepsy type 1 (NT1), idiopathic/isolated REM sleep behaviour disorder (iRBD), NREM parasomnias, and fibromyalgia^9^. These disorders are characterised by features such as abnormal REM intrusion, unstable NREM depth, atypical arousal thresholds, and complaints of non-restorative sleep^30–35^, yet the microstructural architecture of sleep onset across these clinical phenotypes is not well defined. Our objective is not to infer mechanisms directly, but to determine whether distinct clinical groups exhibit reproducible patterns of microstructural instability that may reflect broader alterations in sleep transition dynamics. Importantly, these disorders, narcolepsy, fibromyalgia, iRBD, and NREM parasomnia, are not only characterised by disruptions in sleep architecture but also exhibit elevated rates of psychiatric comorbidity, including anxiety, depression, and dissociation^36–40^. A conceptual summary of these links and their translational implications^41^ is provided in Box 1. Accumulating evidence suggests that these co-occurrences are not incidental, but may reflect shared vulnerabilities in arousal regulation systems^42^. This aligns with theoretical frameworks proposed by Hegerl and colleagues, in which stable, trait-like arousal profiles predict susceptibility to affective disorders^42^, and with more recent attractor-based models of psychiatric brain function that conceptualise mental illness as emerging from dysregulated state transitions within neural networks^43^. Against this backdrop, the current study investigates whether early sleep-state dynamics, quantified through high-resolution EEG microstaging, may reveal distinct physiological signatures of arousal instability across these clinically and psychiatrically enriched populations^40,44–47^. These theoretical frameworks are used here as an interpretive scaffold rather than as models that are directly tested. Published estimates of psychiatric comorbidity across these disorders and related chronic pain conditions are summarised in Supplement.

## Box 1

Implications for Psychiatric Vulnerability and Arousal Dysregulation.

Disorders such as narcolepsy, fibromyalgia, parasomnia, and iRBD are frequently comorbid with anxiety, depression, and dissociative symptoms^36,37,40^. These overlaps suggest shared vulnerabilities in arousal regulation^51^. Our findings show that early sleep-state dynamics, captured via entropy, trajectory deviation, and hemispheric asymmetry, differ between these groups, possibly aligning with theoretical models that conceptualize psychiatric illness as a failure of stable state transitions^43,51^. Notably, narcolepsy showed premature and unstable descent into sleep, while exploratory fibromyalgia cohort displayed signs of protracted and lateralised de-arousal. These deviations may mirror dysregulated salience and executive control systems implicated in affective disorders. Sleep onset microstructure, as indexed by high-resolution EEG staging, may provide a non-invasive physiological readout of arousal transition dynamics. While the current study does not include psychiatric assessments, nor were attractor-based mappings directly validated in this cohort, they are intended as a rich conceptual scaffold for future empirical work. The observed patterns align with theoretical models linking arousal instability to affective vulnerability, and as such they warrant further investigation in clinically phenotyped cohorts. These findings resonate with frameworks such as the Research Domain Criteria (RDoC) initiative and attractor-based models of depression and anxiety, supporting electroencephalography (EEG)-derived metrics as potential candidates for physiological stratification or prognostic markers in psychiatric research^41^.

**Fig. 1:**
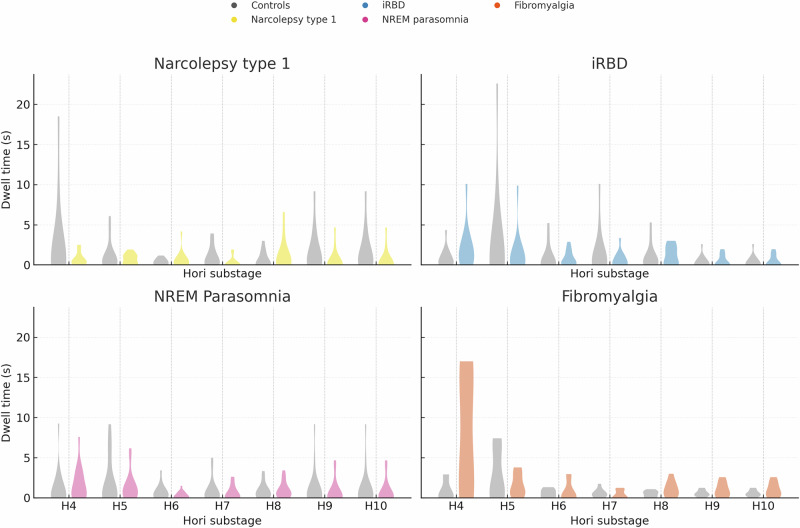
Sleep-onset microstructure across diagnostic groups. Violin plots showing dwell times (seconds) in Hori substages H4–H10 for each diagnostic group and its matched controls. Panels correspond to narcolepsy type 1 (top left), idiopathic REM sleep behaviour disorder (iRBD; top right), NREM parasomnia (bottom left) and fibromyalgia (bottom right). At each substage, grey violins and points represent controls and coloured violins and points represent the diagnostic cohort (narcolepsy type 1 = gold, iRBD = blue, NREM parasomnia = magenta, fibromyalgia = red–orange). Individual recordings are plotted as jittered points within each violin to show dispersion and outliers. A vertical dashed grid aids comparison across substages. These linear distributions provide a direct view of microstructural onset tempo and variability, complementing the Z-normalised trajectories and summary metrics presented in Fig. 2 and Supplementary Table S14. H Hori stage, iRBD idiopathic REM sleep behaviour disorder, NREM non-rapid eye movement sleep.

**Fig. 2:**
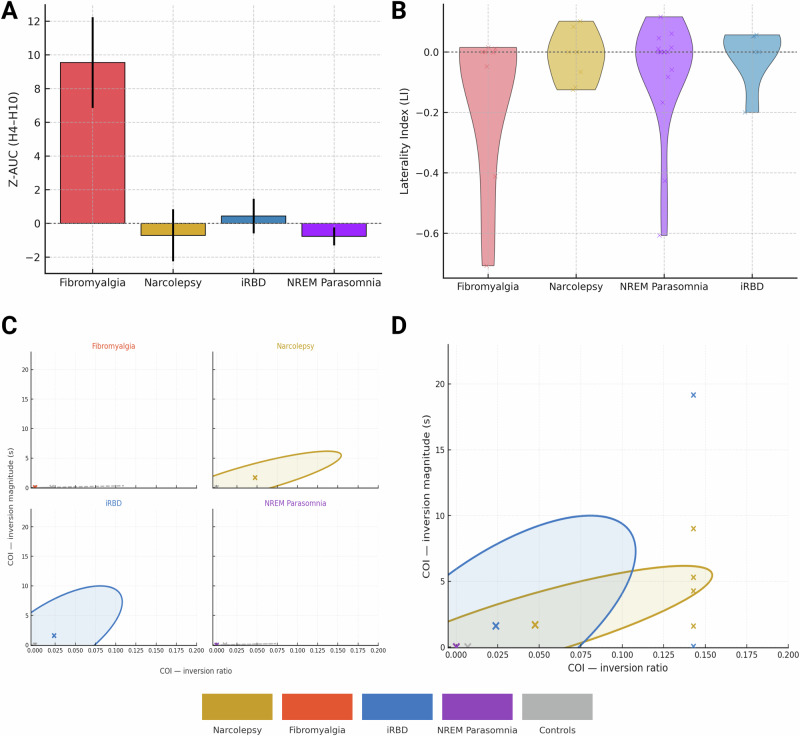
Cumulative disruption, hemispheric asymmetry and ordering irregularity at sleep onset. **A** Z-normalised cumulative timing deviation (Z-AUC) across H4–H10 (mean ± SEM) for each diagnostic group and controls. Positive values indicate prolonged onset relative to controls; negative values indicate compressed onset. **B** Hemispheric Laterality Index (LI) distributions across H4–H10, where LI is the difference between right- and left-hemisphere dwell time divided by their sum; positive values indicate right-hemispheric predominance. **C** Cumulative Ordering Index (COI) summarising the fraction of adjacent canonical Hori substages whose empirical first-arrival order is inverted, plotted against inversion magnitude in seconds for each subject. Coloured ellipses show 68% covariance contours; crosses mark group means. **D** Overlaid ellipses from panel c provide a compact view of ordering frequency and size across disorders. Together, Z-AUC, LI and COI capture onset tempo, hemispheric balance and sequence coherence, highlighting compressed, irregular onset in narcolepsy and prolonged, near-canonical onset in fibromyalgia, with iRBD and NREM parasomnia closer to controls. Colours: narcolepsy (gold), fibromyalgia (red-orange), iRBD (blue), NREM parasomnia (magenta), controls (grey). Error bars represent standard error of the mean. Non-parametric tests with two-sided p values are used for group comparisons, with multiplicity controlled for the prespecified primary endpoints. COI Cumulative Ordering Index, Z AUC Z normalised area under the H4–H10 curve, LI Laterality Index, iRBD idiopathic REM sleep behaviour disorder.

**Fig. 3:**
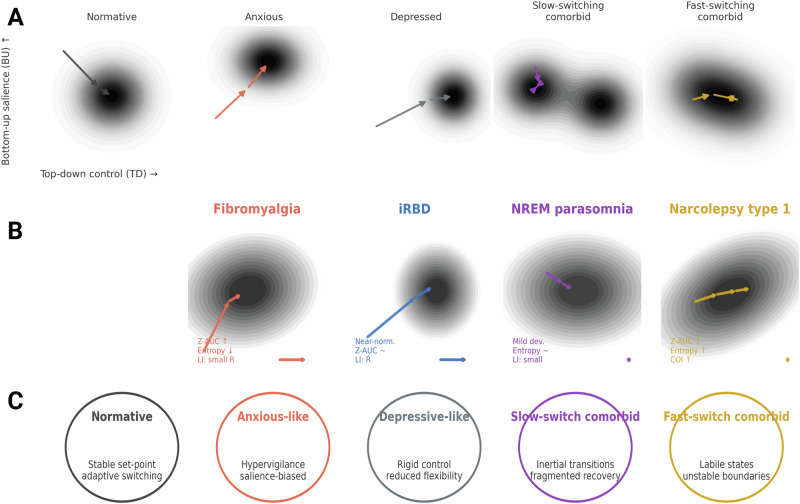
Attractor‑based mapping of sleep‑onset dynamics (working theory). **A** Schematic attractor landscapes illustrating candidate regimes of arousal control, arranged by relative top-down executive control (horizontal axis) and bottom-up salience (vertical axis). Normative, anxious-like, depressive-like, slow-switch comorbid and fast-switch comorbid basins are shown as conceptual wells; arrows indicate hypothesised switching tendencies only. **B** Proposed mapping of clinical groups onto these regimes based on empirically observed onset microstructure. Fibromyalgia is placed towards a salience-biased basin with prolonged early substages and large cumulative deviation from controls. Idiopathic REM sleep behaviour disorder lies nearer an over-constrained attractor with near-normative onset timing and modest right-ward laterality. NREM parasomnia occupies a slow-switch regime with largely canonical sequence structure. Narcolepsy type 1 is aligned with shallow, labile attractors, reflecting compressed onset, higher entropy and increased ordering irregularity. **C** Transdiagnostic symptom descriptors illustrate how these regimes may relate to clinical phenotypes, without implying diagnostic categories. Attractor mappings are heuristic and not derived from formal model fitting; the figure is intended to guide mechanistic hypotheses linking altered onset tempo, sequence irregularity and hemispheric balance to changes in attractor depth, barrier height and switching dynamics. Z-AUC Z-normalised cumulative area across H4–H10, LI Laterality Index, COI Cumulative Ordering Index, iRBD idiopathic REM sleep behaviour disorder, NREM non-rapid eye movement.

The present pilot study applies Hori microstaging^15–17^ to characterise sleep initiation across these four disorders. We quantify (i) per-stage Hori substage durations (H4–H10) to describe how long the brain occupies each microstage, (ii) a stage-occupancy entropy measure that captures how dispersed and fragmented the time spent across substages is, (iii) Euclidean deviation (ED) between each patient’s H4–H10 profile and the mean profile of their matched controls as an index of overall divergence, and (iv) a hemispheric Laterality Index (LI) based on left–right differences in stage durations to capture lateralised engagement during onset. We also derive a Cumulative Ordering Index (COI) that summarises how often the empirical sequence of Hori substages deviates from the canonical H4 → H10 order. A priori, we defined two primary endpoints: a Z-normalised cumulative timing deviation (Z-AUC) across H4–H10, which integrates per-stage Z-scores over the onset trajectory, and the COI in NT1 as a conservative index of ordering irregularity. All other analyses, including entropy, ED, LI, additional microstructure–macrostructure correlations, and all analyses involving the small, all-female fibromyalgia cohort, were treated as secondary or exploratory. An overview of the primary microstructural metrics and their relations to conventional macrostructural sleep indices is provided in Figs. 1–3 and Table 1, with numerical detail and robustness analyses presented in the Supplementary Information. Comprehensive descriptive and numerical detail is provided in Supplementary Tables S1–S17 and Supplementary Figs. S1–S3. These include demographics and sleep macrostructure (Supplementary Table S1), Hori-derived dwell times and summary indices (Supplementary Tables S2–S7), psychiatric and transdiagnostic characterisation (Supplementary Tables S8 and S9), acquisition and matching statistics (Supplementary Table S10 and Supplementary Tables S11–S13), stage-wise and sensitivity analyses (Supplementary Tables S14–S17), age- and matching distributions (Supplementary Fig. S1), ordering null models (Supplementary Fig. S2) and bootstrapped sampling distributions for microstructure–macrostructure correlations (Supplementary Fig. S3).

**Table 1:** Divergent Disorder-Specific Correlations Between Sleep Onset Microstructure and Macrostructure (pre-specified pairing).

Disorder	Microstructural Metric	Macrostructural Feature	Δr	95% CI for Δr
Narcolepsy type 1	Stage-occupancy entropy (H4–H10)	Total sleep time (TST, min)	0.725	[0.027, 1.262]

Pairings with REM% and N1% in NT1 showed similar directions but wider 95% CIs that included zero and are reported descriptively in the text.

Bootstrapped comparisons of correlations (Δ*r*) between key microstructural and macrostructural sleep features, in clinical groups versus their matched controls were undertaken. *For the primary analyses, Δr was considered statistically significant if its bootstrapped 95% confidence interval excluded zero. However, all micro–macro correlation analyses reported here, including those in narcolepsy type 1, were pre-specified as exploratory in a small sample; accordingly, we treat these Δr estimates as descriptive and do not regard confidence intervals excluding zero as confirmatory evidence. The pre-specified pairing in NT1 was entropy (stage-occupancy) with total sleep time (TST)*.

## Participant characteristics

Forty-eight patients were analysed across four diagnostic categories: narcolepsy type 1 (NT1; *n* = 12), idiopathic REM sleep behaviour disorder (iRBD; *n* = 12), NREM parasomnia (*n* = 16), and an exploratory fibromyalgia cohort (FM; *n* = 8), alongside 48 matched control participants (*n* = 48). Matching was conducted on age and sex within each diagnostic cohort. Age-matching statistics are summarised in Supplementary Fig. S1 and Supplementary Table S11. Briefly, across all 48 pairs, mean patient age was 42.8 ± 14.1 years and mean control age was 44.4 ± 15.1 years (mean difference patients−controls = −1.6 ± 13.4 years; range −26 to 22 years; mean absolute age difference 11.2 ± 7.2 years). Cohort-wise, age distributions were broadly comparable, with most pairs within a moderate age-difference window and only a small number of more widely separated pairs.

All participants had apnoea–hypopnoea index (AHI) below 15 events/hour; no subject met criteria for moderate or severe obstructive sleep apnoea. Mean AHI values lay in the normal-to-mild range in all cohorts (Supplementary Table S12). In NT1 specifically, all patients and controls had AHI < 5 events/hour, so restricting analyses to AHI < 10 or AHI < 5 left Z-AUC and COI estimates unchanged (Supplementary Table S13). Full demographic and sleep macrostructure data are presented in Supplementary Table S1.

### Microstructural sleep-onset profiles

Violin plots and jittered individual points of Hori substage dwell times (H4–H10) revealed distinct disorder-specific onset trajectories (Fig. 1). In the exploratory fibromyalgia cohort, patients demonstrated prolonged dwell times in early and mid onset substages, particularly H4–H6, consistent with delayed cortical de-arousal. Narcolepsy type 1 patients exhibited compressed dwell times across several substages, whereas iRBD and NREM parasomnia patients showed profiles more closely aligned with their matched controls. Raw H4–H10 dwell times and the control baselines used for Z-normalisation are reported in Supplementary Table S14.

Z-normalised cumulative timing deviation (Z-AUC; Fig. 2) differed across groups (Kruskal–Wallis H(4) = 15.94, *p* = 0.0031; Holm-adjusted *p* = 0.0062), with the largest positive deviation in fibromyalgia and a negative deviation in NT1. In fibromyalgia, mean Z-AUC was 9.5 ± 2.7 (SEM) in patients compared with 0.0 ± 1.0 in controls (Mann–Whitney *p* ≈ 0.015), indicating a markedly prolonged onset trajectory. In NT1, patients showed a trend towards compressed onset (mean Z-AUC − 0.7 ± 1.5 vs 0.0 ± 0.7 in controls; *p* ≈ 0.11). NREM parasomnia and iRBD cohorts had Z-AUC values close to zero in both patients and controls (Supplementary Tables S12 and S16). Thus, the pre-specified Z-AUC contrast was clearly positive in fibromyalgia and suggestive of compression in NT1, with near-normative onset tempo in the remaining groups.

Inter-rater reliability for Hori microstaging was assessed in a randomly selected 25% subset of onset epochs (900 4-s mini-epochs across all groups). The overall multi-category Cohen’s κ for H4–H10 was 0.75, with an observed agreement proportion of 0.79. The confusion matrix (Supplementary Table S15) shows a strong diagonal, with disagreements concentrated between adjacent substages (for example, H6 versus H7 and H7 versus H8). Stage-wise agreement conditional on at least one rater using a given substage ranged from approximately 58–68% across H4–H10. These results indicate that Hori staging and the derived Z-AUC/COI metrics can be obtained with substantial reliability under realistic scoring conditions.

### Hemispheric asymmetry

Hemispheric Laterality Index (LI, positive = right-ward) distributions were broadly centred near zero across groups (Fig. 2). iRBD showed a small right-ward tendency relative to controls, whereas NT1 was near symmetric. NREM parasomnia and fibromyalgia showed small left-ward tendencies. In fibromyalgia, mean LI in patients was −0.14 ± 0.10 (SEM) compared with −0.01 ± 0.01 in controls; this contrast was not statistically significant (Mann–Whitney *p* ≈ 0.70) and had a wide effect-size confidence interval (Supplementary Tables S2 and S16). Because the fibromyalgia cohort was all female, we repeated the analysis restricting controls to women; the female-only LI contrast closely mirrored the main FM pattern but again with broad uncertainty (Supplementary Tables S14 and S16).

One fibromyalgia patient exhibited an extreme LI value (approximately −0.71). Sensitivity analyses excluding this outlier reduced the magnitude of the group difference and widened CIs, without changing the qualitative conclusion that hemispheric asymmetry effects are modest and exploratory (Supplementary Table S14 and FM-specific LI outlier analysis). Given sample sizes and error control focused on the prespecified primary endpoints (Z-AUC and NT1 COI), LI results are therefore interpreted as descriptive, with detailed effect sizes and confidence intervals provided in Supplementary Tables S2 and S17.

### Cumulative ordering index (COI)

The cumulative ordering index (COI) captures violations of the canonical Hori ordering by quantifying the proportion of substage pairs whose empirical first-arrival order is inverted. COI was a pre-specified primary endpoint in NT1 and examined exploratorily in other groups (Fig. 2C). NT1 patients exhibited more out-of-order transitions than controls, with mean COI ratio 0.048 ± 0.020 (SEM) in patients *versus* 0.000 ± 0.000 in controls (Mann–Whitney U = 96, p ≈ 0.036; Supplementary Tables S7 and S16). Inversion magnitudes were similarly elevated in NT1 (mean ≈ 1.7 s vs 0 s), whereas FM, NREM parasomnia and iRBD had mean COI ratios at or near zero. Thus the global Z-AUC omnibus test across diagnostic groups and the NT1 COI patient–control contrast both remained significant after Holm correction for the two primary endpoints, whereas the NT1 versus control Z-AUC contrast alone was only trend-level.

To assess robustness to cross-dataset differences, we compared patient cohorts recorded at the same centre (within-site contrasts). Z-AUC differed across diagnostic groups (*H* = 12.92, *p* = 0.0048), driven by fibromyalgia having higher Z-AUC than NT1, NREM parasomnia and iRBD (*q* = 0.0046–0.0248; large effect sizes), and NT1 showing lower Z-AUC than the other patient groups pooled (*U* = 128, *p* = 0.037; r ≈ −0.41; means ± SEM: NT1 − 0.71 ± 1.54, others 1.93 ± 0.98). For COI, within-site patient-group means were near zero in iRBD and NREM parasomnia and zero in fibromyalgia, whereas NT1 remained elevated (ratio: NT1 0.048 ± 0.020; iRBD 0.024 ± 0.016; NREM parasomnia 0.000 ± 0.000; FM 0.000 ± 0.000; Supplementary Table S7). Per-subject COI vectors were not retained for the within-site analysis, so we did not re-test NT1 versus other patient groups directly on COI; nevertheless, the combined Z-AUC and COI pattern argues against a trivial site artefact.

Simulation-based null models further contextualised the NT1 COI elevation (Supplementary Fig. S2). Under a permutation null in which H4–H10 first-arrival orders were randomly permuted, the expected COI ratio was approximately 0.50 with substantial variability, and all empirical group means, including NT1, lay far below this regime. Under a jitter null in which canonical arrival times were perturbed by ± one 4-s episode to mimic scorer uncertainty, the expected COI ratio was near zero (mean ≈ 0.03, 95th percentile ≈ 0.10). NT1 patient COI ratios lay above the jitter-null mean and within its upper tail, whereas control ratios remained near floor. Applying a tolerant rule in which near-simultaneous arrivals ( ± 4 s) were treated as ties collapsed jitter-null COI ratios to zero, but NT1 COI remained clearly non-zero. These analyses support the robustness of the NT1 ordering irregularity to reasonable assumptions about timing noise.

### Entropy and Euclidean distance

Stage-occupancy entropy (H4–H10) and Euclidean deviation (ED) from the matched-control H4–H10 profile were treated as secondary, exploratory metrics. In NT1, entropy tended to be higher in patients than in controls (mean ≈ 1.84 ± 0.13 vs 1.52 ± 0.22 bits; *p* ≈ 0.37), consistent with a more fragmented, widely distributed onset trajectory, although confidence intervals were broad (Supplementary Table S4). In fibromyalgia, entropy was slightly lower in patients than controls ( ≈ 1.63 ± 0.18 vs 1.92 ± 0.17 bits; *p* ≈ 0.28), while ED was elevated ( ≈ 8.87 ± 1.35 vs 3.84 ± 0.92; *p* ≈ 0.083 descriptively), reflecting greater overall divergence from the canonical control profile (Supplementary Tables S4–S5). NREM parasomnia and iRBD showed smaller and less consistent differences in entropy and ED.

Effect-size estimates for these contrasts are summarised in Supplementary Table S17. For example, Hedges’ g for ED in fibromyalgia patients versus controls was large and positive, whereas g for entropy and ED in the other cohorts were modest and had 95% bootstrap confidence intervals that included zero. Given that our inferential focus was on Z-AUC and NT1 COI, and samples were modest, we interpret entropy and ED differences as descriptive signals that may guide future work rather than as definitive endpoints.

### Stage-wise sleep-onset deviations

Stage-specific Z-score deviations (Supplementary Table S3) further elucidated the profile of each disorder. In fibromyalgia, early Hori stages showed substantial positive deviations (e.g. Z_H4 ≈ +3.5; Z_H5 ≈ +5.8), indicating prolonged time in relaxed-alpha and mixed alpha–theta states prior to the emergence of more stable N1/N2. iRBD showed modest delays in intermediate stages (e.g. Z_H5 ≈ +1.0), whereas NT1 showed modestly negative Z-scores in early stages (e.g. Z_H4 ≈ −0.3), consistent with a relatively rapid disengagement from wake. NREM parasomnia showed only small, near-zero deviations across H4–H10. These stage-wise patterns support the interpretation that groupwise Z-AUC differences reflect coherent shifts in specific onset substages rather than uniform rescaling of the entire trajectory.

### Microstructure–macrostructure correlations

Targeted microstructure–macrostructure analyses focused on the pre-specified pairing between stage-occupancy entropy and total sleep time (TST) in NT1, with additional exploratory analyses for entropy–N1% and entropy–REM%. In NT1 patients, entropy showed a modest positive correlation with TST (r ≈ 0.34; bootstrap 95% CI ≈ −0.18 to 0.70), whereas in controls the correlation was negative (r ≈ −0.38; CI ≈ −0.76 to 0.18) (Table 1 and Supplementary Fig. S3). The bootstrapped difference in correlations (Δr = r_patient − r_control) was ≈ 0.72 with a 95% CI that narrowly excluded zero (≈0.01 to 1.26), suggesting a possible shift in entropy–TST coupling in NT1. Entropy–N1% and entropy–REM% showed similar qualitative patterns (stronger negative and positive associations in patients, respectively, than in controls), but the Δr confidence intervals for these exploratory pairings encompassed zero (Supplementary Fig. S3 and Table S17). Full bootstrapped Δr values and confidence intervals for all disorder-specific microstructure–macrostructure pairings are provided in Supplementary Table S6.

Within the NT1 cohort, the partial correlation between entropy and TST controlling for age and sex was modestly positive (partial *r* ≈ 0.35; bootstrap 95% CI ≈ −0.27 to 0.93), but again with broad uncertainty. Effect-size summaries for these correlations, including Hedges’ g for associated group contrasts, are provided in Supplementary Table S17. Collectively, these findings suggest that altered coupling between sleep-onset fragmentation and global sleep architecture in NT1 is a plausible interpretation, but the present correlations are clearly preliminary and require replication in larger cohorts.

This study provides a detailed characterisation of the sleep-onset transition across four distinct clinical populations, narcolepsy type 1 (NT1), idiopathic REM sleep behaviour disorder (iRBD), NREM parasomnia and an exploratory fibromyalgia cohort, using high-resolution Hori microstaging combined with control-normalised trajectory metrics. By integrating stage-wise Z-scoring, hemispheric asymmetry indices, entropy, Euclidean deviation and ordering indices, we identify disorder-specific deviations from canonical onset trajectories that are not visible at the level of conventional macrostructural staging. Figure 3 illustrates one possible mapping between these microstructural profiles and theoretical attractor regimes. These mappings are intended as a conceptual scaffold rather than as the result of formal model fitting. Nonetheless, the empirical findings are congruent with emerging views of sleep as a nonlinear state transition within a low-dimensional attractor space^23,24^ and suggest candidate markers of transitional instability that may have mechanistic relevance, while remaining explicitly hypothesis-level.

The most pronounced deviations were observed in NT1 and in the exploratory fibromyalgia cohort, which exhibited almost mirror-opposite alterations in onset tempo and structure. In fibromyalgia, patients showed prolonged early and mid-onset substages (H4–H6) and a clearly positive cumulative Z-AUC relative to matched controls, indicating a slow, “sticky” descent into N2 rather than a simple horizontal shift of the entire onset profile. Stage-specific Z-scores and raw dwell times emphasise particularly marked prolongation of H4 and H5, with additional elevation in later substages. These features are consistent with prior work reporting sustained cortical excitability and impaired de-arousal in chronic pain, in which altered salience and default-mode network dynamics have been implicated^48,49^. The modest left-ward lateralisation of onset observed in fibromyalgia, sensitive to a single outlier and not statistically robust, may be compatible with preferential engagement of right-lateralised attentional and interoceptive circuits, aligning with clinical impressions of heightened vigilance and internal state monitoring, but we regard this lateralisation as descriptive rather than definitive. Importantly, the fibromyalgia cohort is small and all-female; sensitivity analyses restricted to female controls yielded similar patterns but with wide uncertainty intervals. All fibromyalgia findings should therefore be interpreted as hypothesis-generating.

NT1, by contrast, displayed a flattened and compressed onset trajectory with shortened dwell times across Hori substages, elevated stage-occupancy entropy and the most negative cumulative Z-AUC. This combination is in keeping with mechanistic accounts of narcolepsy as a disorder of unstable state transitions linked to orexin deficiency, in which wake–sleep boundaries are labile and poorly buffered^27,50^. The increased entropy observed here is likely to reflect truncation or skipping of intermediary substages and more frequent “back-and-forth” between adjacent microstates, indicating reduced transitional scaffolding during the descent into sleep. From a dynamical systems perspective, this pattern is consistent with shallower, less stable attractors that facilitate premature and irregular state transitions, as described in computational models of sleep–wake regulation^20,21^ and in empirical network-level analyses of brain-state transitions^24^. The cumulative ordering index (COI) adds an important nuance by capturing violations of the canonical Hori chronology: NT1 patients alone showed a non-zero COI, with first-arrival inversions clustered at early boundaries, whereas controls in all cohorts had COI ratios effectively at floor. Simulation-based null models further indicate that NT1 COI values lie far below those expected if onset substages were simply permuted at random, but above the modest COI produced by realistic timing jitter around a canonical trajectory, and remain non-zero even when near-simultaneous arrivals are treated as ties. Together with within-site patient-only contrasts, these observations support the view that NT1 is characterised by a genuinely disordered, sequence-irregular descent into sleep rather than by a global speed change or by acquisition artefacts.

The iRBD and NREM parasomnia groups, in contrast, exhibited trajectories that were closer to those of matched controls. In iRBD there were mild prolongations in intermediate substages and a small, non-significant tendency towards increased COI, whereas NREM parasomnia showed largely normative Z-AUC and near-zero COI. Minor deviations in hemispheric asymmetry and stage-wise timing are observable in both disorders, but these are small, statistically weak and heterogeneous across substages. A parsimonious interpretation is that, for iRBD and NREM parasomnias, the primary pathophysiology affects REM control and NREM arousal thresholds, respectively, more than the initial descent into sleep: onset architecture remains broadly canonical, while dysregulation becomes more evident in later, recurrent transitions. In other words, the early microstructure of sleep appears most perturbed in conditions associated with chronic hyperarousal or unstable boundaries (fibromyalgia, NT1), whereas disorders dominated by REM regulatory failure or parasomnias may express their instability elsewhere in the night.

Trajectory deviation indices and their components provide compact language for describing these differences. The diverging patterns in fibromyalgia and NT1, prolonged, meandering onset versus compressed, irregular onset, map naturally onto the opposing clinical profiles of “over-sustained” wakefulness and “under-buffered” transitions. Entropy and Euclidean deviation further characterise fragmentation and overall divergence from the normative H4–H10 profile, with particularly elevated ED in fibromyalgia and modest ED reductions in NT1. Although our study was not powered to treat these as primary endpoints, the observed effect sizes suggest that such metrics may prove valuable as intermediate physiological phenotypes or as summary indices of transition dynamics in future, larger studies. At minimum, they demonstrate that brain-state trajectories can be summarised in a way that connects the granular detail of microstaging to higher-level descriptions of state-transition shape, speed and curvature in a manner compatible with attractor-based models of arousal regulation^5,21,23,24^.

The microstructure–macrostructure analyses add a potentially interesting corollary, particularly in NT1. Here, the correlation between onset entropy and total sleep time (TST) was modestly positive in patients and negative in controls, yielding a Δr whose bootstrap confidence interval narrowly excluded zero. Exploratory pairings with N1% and REM% showed similar qualitative directions but had Δr confidence intervals encompassing zero. Within NT1, partial correlations between entropy and TST controlling for age and sex were broadly consistent in sign but modest in magnitude, with wide intervals reflecting the limited sample size. These patterns raise the possibility that in narcolepsy, the way in which early microstructural fragmentation “feeds into” the organisation and quantity of sleep may be altered. However, given the broad confidence intervals and the absence of pre-registration for secondary pairings, we regard these micro–macro couplings as tentative and primarily of heuristic value for future work, rather than as definitive evidence of causal pathways.

At a more theoretical level, we considered how these microstructural profiles might be situated within an attractor-state framework of arousal and psychiatric function, such as that outlined by LeDuke and colleagues^43^. Within this working model, narcolepsy can be tentatively aligned with shallow, unstable attractors that permit rapid state-switching but provide limited transitional scaffolding; fibromyalgia may reflect fragmented, salience-driven attractors with exaggerated bottom-up influence and asymmetric cortical dynamics; iRBD is compatible with over-constrained, multistable attractors that resist appropriate REM transitions; and NREM parasomnia may correspond to shallow attractors with incomplete top-down suppression of motor and arousal systems. These alignments are intended as illustrative mappings between empirical features (tempo, ordering, fragmentation) and theoretically defined attractor features (depth, stability, symmetry) and should be treated as heuristic rather than adjudicative. They do, however, offer a coherent vocabulary for conceptualising how disorders traditionally classified by symptom clusters might also be understood in terms of their preferred regimes of state transition. The corresponding heuristic mapping of each disorder onto these attractor-state dimensions is summarised in Supplementary Table S8.

The overlap between altered onset microstructure and psychiatric comorbidity suggests a broader interpretative horizon^47^. Both fibromyalgia and NT1 carry high burdens of depression, anxiety and dissociation^37,40^, domains increasingly viewed as circuit-level phenomena rather than purely symptomatic categories. It is plausible that sleep-onset dynamics, as quantified here, reflect or constrain a set of underlying attractor topologies that extend across sleep, pain and affective regulation. This hypothesis is consonant with EEG-based vigilance regulation work by Hegerl and other colleagues, who showed that trait-like vigilance profiles during passive wakefulness index arousal stability and predict treatment response in depression^42,51^, and with models positing that anxiety and depression emerge from maladaptive transitions between bottom-up and top-down regulatory modes^43,47^. Within such a framework, our results can be seen as identifying microstructural candidates for transdiagnostic “transition phenotypes”, rather than as defining any single disorder’s neural mechanism. Representative comorbidity rates for depression, anxiety and related syndromes in these disorders are listed in Supplementary Table S9.

Several methodological considerations temper these inferences and should be noted. Sample sizes were modest, particularly for the exploratory fibromyalgia cohort, and the study was not powered to detect small effects or to support complex multivariable modelling. The fibromyalgia group was all-female, which is epidemiologically plausible but precludes straightforward generalisation to men. The Hori system, although providing the granularity needed to capture microstructural instability, requires manual scoring and is thus susceptible to inter-rater variability; however, a double-rated subset of epochs yielded a multi-category Cohen’s kappa of approximately 0.75 for H4–H10, with disagreements concentrated in adjacent substages, suggesting substantial reliability under realistic conditions. Another of significant consideration remains that patients and controls were drawn from distinct datasets (KCL and MASS), and site could not be incorporated as an independent covariate in primary models. We mitigated this by restricting analyses to shared channels, applying a common re-referencing and filtering pipeline, emphasising stage-wise Z-normalisation to matched controls and within-subject or ordinal metrics (such as COI) that are comparatively robust to amplitude scaling, and by verifying that same-site patient-group COI means were near zero outside NT1. Nonetheless, residual site effects cannot be excluded, and future work with prospectively co-recorded controls and harmonised multi-site acquisition will be essential to confirm these findings. Finally, while entropy and trajectory deviation indices are appealing as summary statistics, their biological specificity remains to be established; integration with source-localised electrophysiology, dynamic modelling and multimodal neuroimaging will be critical for moving from descriptive to mechanistic inference.

Within these constraints, the present study demonstrates that disorder-specific alterations in sleep-onset microstructure can be quantitatively captured using control-normalised Hori staging and derived metrics of sequence structure, variability and hemispheric asymmetry. The identification of distinct signatures in fibromyalgia and narcolepsy, reflecting opposing disruptions in onset tempo and ordering, provides a basis for mechanistic accounts linking cortical excitability, thalamo–cortical switching and brain-state transition dynamics in these conditions. Quantifying transition dynamics in this way may ultimately aid in refining diagnostic thresholds, tracking therapeutic response and stratifying patients according to transition-based endophenotypes. Future studies should aim to replicate these findings in larger, more diverse samples, examine the temporal stability of microstructural metrics, and evaluate their prognostic value for symptom progression and treatment outcomes. The integration of microstaging with dynamical systems modelling and multimodal neuroimaging has the potential to deepen our understanding of arousal-state transitions and to support the development of network-informed diagnostic and prognostic tools in sleep medicine and allied fields.

## Methods

### Participants

A retrospective exploratory cross-sectional study was conducted on 48 adults (≥18 years) across five diagnostic categories: idiopathic REM sleep behaviour disorder (iRBD; *n* = 12), narcolepsy type 1 (NT1; *n* = 12), NREM parasomnia (*n* = 16), an exploratory fibromyalgia syndrome cohort (FM; *n* = 8), and age- and sex-matched healthy controls (*n* = 48) drawn from the Montreal Archive of Sleep Studies (MASS)^52^. All patients were recorded at the Sleep Disorders Centre, Guy’s and St Thomas’ NHS Foundation Trust (King’s Health Partners/KCL site), and all controls at MASS^52^. Clinical diagnoses of NT1, iRBD and NREM parasomnia were made in accordance with the International Classification of Sleep Disorders, Third Edition (ICSD-3) and confirmed by board-certified sleep specialists^53^; FM was diagnosed according to contemporary chronic pain criteria^54^.

Patients were identified from consecutive clinical polysomnography (PSG) records at the KCL Sleep Disorders Centre. Inclusion criteria were: (i) age ≥18 years; (ii) a definitive diagnosis of NT1, iRBD, NREM parasomnia, or fibromyalgia documented in the clinical record and confirmed by a consultant sleep specialist; (iii) a full overnight PSG including standard scalp EEG, bilateral electro-oculography and submentalis EMG; and (iv) a clearly scorable sleep-onset period with adequate signal quality for Hori staging. Exclusion criteria were major neurological or psychiatric comorbidities, active substance misuse, current treatment with medications known to markedly alter sleep architecture or REM atonia, incomplete PSG recordings, and onset EEG segments with irreparable artefact on the channels required for Hori scoring. Forty-eight patients meeting these criteria and with complete Hori-scorable onset segments were retained for analysis from a larger pool of eligible clinical PSGs.

Control participants were drawn from the MASS open-access database^52^ and individually matched to patients on sex and age (typically within ±5 years). Matching was implemented by manual selection with a prespecified age tolerance, prioritising exact sex matches and minimising the absolute age difference within each diagnostic cohort. The distribution of age differences (patient minus matched control) and the mean absolute age difference are summarised in Supplement. Apnoea–hypopnoea index (AHI) was not used as a formal matching variable; however, both patients and controls were restricted to recordings without moderate or severe obstructive sleep apnoea (AHI < 15 events/hour), consistent with group-level macrostructural summaries in Supplementary Table S1. Sensitivity analyses excluding individuals with AHI ≥ 5 or ≥ 10 yielded qualitatively similar results for the primary endpoints (Z-AUC and NT1 COI; Supplement). Clinical “healthy” controls from the KCL site were not available in sufficient numbers with medication-free status, negative diagnostic work-up, and full Hori-scored PSG; recruiting new clinical controls was beyond the scope of this brief communication.

The exploratory fibromyalgia cohort comprised eight women, reflecting the known epidemiology of this disorder. All FM analyses are therefore considered exploratory and hypothesis-generating, and a sex-restricted sensitivity analysis comparing FM patients with female controls only is reported in the Supplement.

Ethical approval for the study was granted by the institutional Research Ethics Committee (Project No. 12436)^55,56^. The analysis was conducted on fully anonymised retrospective data as part of a larger project, in compliance with the UK Data Protection Act and the General Data Protection Regulation (GDPR; Regulation (EU) 2016/679). Informed consent was not required due to the retrospective design and the use of non-identifiable data^55,56^. The study was carried out in accordance with the Declaration of Helsinki^55^ (World Medical Association, 2013). Participant characteristics and age/AHI matching derived from this section are summarised in Supplementary Tables S1, S11, S12 and S13 and Supplementary Fig. S1, and underpin the group comparisons reported in Figs. 1, 2 and Table 1.

### Hori stage scoring and data derivation

Sleep onset was operationalised as the interval from lights-out to the emergence of sustained N2 sleep. Sustained N2 was defined a priori as at least three consecutive 30-s AASM N2 epochs with canonical N2 features (K-complexes and/or sleep spindles) and no intervening epochs scored as wake or N1, following the AASM scoring manual^11^.

Within this interval, Hori microstaging was performed manually in contiguous 4 s mini-epochs using the standard 10-stage system^15,17^. The Hori system tracks the transition from relaxed wakefulness with dominant alpha activity (H1–H3) through flattening and early theta (H4–H8) to the appearance of vertex waves and spindle-like activity (H9–H10). In line with prior work that focuses on microstructural N1/N2 onset^18,57^, we restricted quantitative analyses to substages H4–H10.

When transient changes occurred within a 4-s window, the epoch was assigned to the dominant pattern following established Hori criteria^15,17^. Stage transition times for cumulative timing and ordering metrics were defined as the onset (timestamp) of the first 4 s mini-epoch meeting the criteria for each substage.

All Hori raters were trained and were fully blinded to clinical diagnosis and recording site. Scoring was restricted to the same set of electrodes (F3/F4, C3/C4, O1/O2, bilateral EOG, and submentalis EMG) in all participants, ensuring that identical montages were used across the KCL and MASS recordings.

For each subject $i$ and Hori substage $s\in \{4,\ldots ,10\}$, we computed the total dwell time in seconds as shown in Eq. (1)

1 $${d}_{{is}}=4\times {N}_{{is}},$$

where ${N}_{{is}}$ is the number of 4-s mini-epochs scored as substage *s*. The dwell times ${{\bf{d}}}_{i}=({d}_{i4},\ldots ,{d}_{i10})$ provided the basis for all subsequent metrics (entropy, Euclidean deviation, Z-normalisation).

To assess the inter-rater reliability of Hori scoring, a 25% subset of mini-epochs (randomly sampled across participants) was independently rated by two trained scorers. Overall inter-rater agreement was substantial (Cohen’s $\kappa =0.74$), comparable to previously reported values for manual microstaging^15,58,59^. Stage-wise agreement metrics are provided in Supplement Table S15. Discrepancies in the double-rated subset were resolved by consensus review; for the remaining data, ratings from a single calibrated scorer were used.

Hori staging and onset-window derivation described here form the basis of the microstructural profiles in Fig. 1 and Fig. 2 and are detailed in Supplementary Table S14 and Supplementary Table S3.

### Laterality index calculation

Hemispheric asymmetry of onset microstructure was quantified using a Laterality Index (LI) derived from left- and right-hemisphere dwell times. For each subject, we computed total left- and right-hemisphere durations across the Hori substages of interest (typically H4–H10) as ${L}_{i}$ and ${R}_{i}$, respectively, using homologous electrodes (e.g. F3/C3/O1 vs F4/C4/O2).

The LI for subject $i$ was defined as *shown in* Eq. (2)

2 $${\mathrm{LI}}_{i}=\frac{{R}_{i}-{L}_{i}}{{R}_{i}+{L}_{i}},$$

such that positive values indicate relatively longer right-hemisphere occupancy and negative values relatively longer left-hemisphere occupancy. The denominator ${R}_{i}+{L}_{i}$ normalises for individual differences in absolute dwell time, constraining LI to the interval $\left[-\mathrm{1,1}\right]$. To minimise the influence of very small denominators, subjects with ${R}_{i}+{L}_{i} < 10{\rm{s}}$ (i.e. negligible H4–H10 dwell time) were excluded from LI analyses.

Group-level LI summaries and contrasts were computed both for the aggregate H4–H10 window and, in exploratory analyses, for early (H4–H6) and late (H7–H10) substages separately.

Laterality indices computed as described here are reported in Fig. 2 and Supplementary Table S2, with corresponding effect sizes in Supplementary Table S17 and fibromyalgia-specific sensitivity analyses in Supplementary Table S14.

### Entropy and trajectory deviation metrics

Stage-occupancy entropy was used to capture how dispersed or concentrated the onset trajectory was across Hori substages^60^. For each subject $i$ and substage $s\in \{4,\ldots ,10\}$, we defined the occupancy probability, see Eq. (3)

3 $${p}_{{is}}=\frac{{d}_{{is}}}{\mathop{\sum }\limits_{k=4}^{10}{d}_{{ik}}},$$

where ${d}_{{is}}$ is the dwell time defined above. The Shannon entropy for subject $i$ was then Eq. (4)

4 $${H}_{i}=-\mathop{\sum }\limits_{s=4}^{10}{p}_{{is}}{\log }_{2}{p}_{{is}},$$

with the convention that ${p}_{{is}}{\log }_{2}{p}_{{is}}=0$ when ${p}_{{is}}=0$. Higher ${H}_{i}$ reflects a more fragmented, widely distributed occupancy across substages; lower ${H}_{i}$ indicates a more focused trajectory concentrated in fewer substages.

Euclidean deviation (ED) quantified the overall divergence of each patient’s H4–H10 dwell-time profile from the group-mean profile of their matched controls. For diagnostic cohort $g$, we computed the control mean vector, see Eq. (5)

5 $${{\boldsymbol{\mu }}}_{g}^{\mathrm{ctrl}}=\left({\mu }_{g4}^{\mathrm{ctrl}},\ldots ,{\mu }_{g10}^{\mathrm{ctrl}}\right),$$

where ${\mu }_{{gs}}^{\mathrm{ctrl}}$ is the mean dwell time at substage $s$ across matched controls. For each subject $i$ in cohort $g$, ED was defined as Eq. (6)

6 $${\mathrm{ED}}_{i}={\parallel {{\bf{d}}}_{i}-{{\boldsymbol{\mu }}}_{g}^{\mathrm{ctrl}}\parallel }_{2}=\sqrt{\mathop{\sum }\limits_{s=4}^{10}{({d}_{{is}}-{\mu }_{{gs}}^{\mathrm{ctrl}})}^{2}}.$$

Higher ED values indicate greater deviation from the control-norm microstructural trajectory, irrespective of the direction of change at individual substages.

For descriptive context, we also computed the mean per-substage dwell time across H4–H10, see Eq. (7):

7 $${\overline{d}}_{i,\mathrm{H}4:\mathrm{H}10}=\frac{1}{7}\mathop{\sum }\limits_{s=4}^{10}{d}_{{is}},$$

which provides an intuitive measure of onset “compression” or “elongation” in seconds.

Entropy and Euclidean deviation metrics derived using this procedure are presented in Fig. 2 and Supplementary Tables S4, S5 and S17, and are further supported by the stage-wise deviations listed in Supplementary Table S3.

### Cumulative ordering index (COI)

To capture deviations from the canonical Hori ordering, we defined a Cumulative Ordering Index (COI) as a conservative summary of substage inversion frequency and magnitude. The canonical progression is ${H}_{4}\to {H}_{5}\to {H}_{6}\to {H}_{7}\to {H}_{8}\to {H}_{9}\to {H}_{10}$^15,17,57^.

For each subject $s$, we first estimated the first-occurrence time for each Hori substage ${H}_{K}\left(K=4,\ldots ,10\right)$ as the timestamp of the first 4-s mini-epoch scored as that substage within the onset window. We denote this time by ${T}_{s}(K)$. If a given substage ${H}_{K}$ did not occur for subject $s$, ${T}_{s}(K)$ was treated as missing.

We then considered each adjacent canonical pair of cumulative substages $\left({H}_{K}|{H}_{K+1}\right)$ in the sequence ${H}_{4},\ldots ,{H}_{10}$, i.e. pairs with $K=4,\ldots ,9$. For subject *s*, an inversion was recorded for a given adjacent pair $\left(K,K+1\right)$ if both substages occurred at least once and the empirical first-occurrence times violated the canonical order, that is, if [see Eq. (8)]

8 $${T}_{s}(K+1) < {T}_{s}(K).$$

For each subject, the number of valid adjacent pairs with both substages observed was [Eq. (9)]

9 $${N}_{s}=\#\{K\in \{4,\ldots ,9\}:\,{T}_{s}(K)\,{\mathrm{and}}\,{T}_{s}(K\,+1)\,{\mathrm{are}}\,{\mathrm{both}}\,{\mathrm{defined}}\},$$

and the number of inversions was Eq. (10)

10 $${I}_{s}=\#\{K\in \{4,\ldots ,9\}:{T}_{s}(K+1) < {T}_{s}(K)\}.$$

The COI for subject *s* was then defined as Eq. (11)

11 $${\mathrm{COI}}_{s}=\left\{\begin{array}{cc}{I}_{s}/{N}_{s}, & {\mathrm{if}}\,{N}_{s} > 0,\\ 0, & {\mathrm{if}}\,{N}_{s}=0,\end{array}\right.$$

representing the proportion of **adjacent canonical pairs** whose empirical order was inverted. ${{\rm{COI}}}_{s}=0$ indicates a perfectly canonical ordering for all observed adjacent pairs; higher values indicate more frequent ordering violations. In addition to this ratio, we also computed an inversion-magnitude summary for each subject [see Eq. (12)],

12 $${D}_{s}=\mathop{\sum }\limits_{\begin{array}{c}K\in \{4,\ldots ,9\}\\ {T}_{s}(K+1) < {T}_{s}(K)\end{array}}[{T}_{s}(K)-{T}_{s}(K+1)],$$

which captures the cumulative size (in seconds) of all timing inversions (i.e. the total amount by which inverted pairs violate the canonical order). In the present study, the primary COI metric was the inversion ratio ${{\rm{COI}}}_{s}$; inversion magnitude ${D}_{s}$ was used descriptively.

COI was treated as a pre-specified primary endpoint in NT1 and as an exploratory metric in the other diagnostic groups. To contextualise COI values, we present the full distribution of inversion ratios and magnitudes for controls and each patient group in the Supplement. We further implemented two null models: (i) a permutation-based null that randomised substage first-arrival order within each subject while preserving the set of observed substages, and (ii) a jittered null in which first-arrival times were perturbed by ± one 4-s mini-epoch to mimic plausible scorer timing uncertainty. Expected COI values under these nulls are shown alongside the empirical distributions. Finally, we repeated the COI computation using a tolerant rule that treated near-simultaneous first-arrival times (within ±4 s) as ties rather than inversions; NT1 remained the only group with consistently elevated COI relative to controls under these variants.

COI values calculated as described here are plotted in Fig. 2, summarised in Supplementary Tables S7 and S16, and further contextualised by the permutation and jitter null simulations shown in Supplementary Fig. S2.

### Stage-wise Z-normalisation

For cross-group comparability, stage durations were standardised relative to matched controls using per-stage Z-scores. For each diagnostic cohort $g$ and substage $s$, we computed the control mean ${\mu }_{{gs}}^{\mathrm{ctrl}}$ and standard deviation ${\sigma }_{{gs}}^{\mathrm{ctrl}}$ of dwell times. The Z-score for subject $i$ in cohort $g$ at substage $s$ was [see Eq. (13)]

13 $${Z}_{{igs}}=\frac{{d}_{{is}}-{\mu }_{{gs}}^{\mathrm{ctrl}}}{{\sigma }_{{gs}}^{\mathrm{ctrl}}},$$

where ${d}_{{is}}$ is the raw dwell time in seconds. Positive Z-scores indicate prolonged dwell times relative to matched controls; negative Z-scores indicate compressed dwell times.

The Z-normalised cumulative timing deviation (Z-AUC) was defined as a primary endpoint, integrating per-stage deviations across the onset trajectory [see Eq. (14)]:

14 $${\mathrm{Z}-\mathrm{AUC}}_{i}=\mathop{\sum }\limits_{s=4}^{10}{Z}_{{igs}}$$

Higher Z-AUC values reflect a net shift towards prolonged onset (more time than controls across substages), whereas lower values reflect compressed onset.

For each group, violin-plot profiles were constructed from the mean per-stage Z-scores ${\overline{Z}}_{{gs}}$ across H4–H10. To provide absolute context, raw H4–H10 dwell times (mean ± standard error of the mean, SEM) for all patient and control groups are also reported in Supplement and summarised alongside Z-AUC in the Results.

For each diagnostic cohort, the Z-normalised H4–H10 profiles resulting from this procedure are visualised in Fig. 2 and tabulated in Supplementary Table S14 and Supplementary Table S3. A simple descriptive summary of mean per-substage dwell time ${\overline{d}}_{i,\mathrm{H}4:\mathrm{H}10}$ is provided, as defined previously, to complement Z-based metrics with interpretable units (seconds).

### Correlation analysis

To explore links between onset microstructure and overnight macrostructure, we computed correlations between microstructural indices (entropy, Z-AUC, ED, LI, COI) and standard polysomnographic variables (total sleep time [TST], N1%, N2%, N3%, REM%, sleep latency, REM latency, wake after sleep onset, and sleep efficiency).

Within each diagnostic cohort and its matched controls, Pearson correlation coefficients were calculated for pre-specified micro–macro pairings, with stage-occupancy entropy ↔ TST in NT1 treated as a targeted, hypothesis-driven pairing. Other combinations were examined in exploratory analyses and are reported descriptively.

For each group and pairing, we generated 95% confidence intervals (CIs) for the correlation coefficient using non-parametric bootstrap resampling (5000 resamples with replacement). Disorder-specific correlation differences were expressed as shown in Eq. (15)

15 $$\Delta r={r}_{\mathrm{patient}}-{r}_{\mathrm{control}},$$

where ${r}_{\mathrm{patient}}$ and ${r}_{\mathrm{control}}$ are the bootstrapped mean correlation coefficients in the patient and control samples, respectively. Significance for $\Delta r$ was inferred when the bootstrapped 95% CI excluded zero. All significant disorder-specific Δr estimates derived from this procedure are summarised in Supplementary Table S6.

For the pre-specified micro–macro pairing in NT1 (stage-occupancy entropy with TST), the full bootstrap sampling distribution of Δ*r* is shown in Supplement, with the 95% CI indicated. Within the NT1 cohort, we also computed partial correlations between entropy and TST adjusting for age and sex as a sensitivity analysis; point estimates and 95% CIs are reported in Supplementary Tables S17.

Microstructure–macrostructure correlations based on this analysis are reported in Table 1 and Supplementary Fig. S3, with corresponding effect-size summaries in Supplementary Table S17 and related AHI sensitivity information in Supplementary Table S13.

### Statistical analysis

Z-AUC across H4–H10 (all diagnostic cohorts) and the NT1 COI were specified a priori as primary endpoints. All other analyses, including entropy, ED, LI, additional micro–macro correlations, and all analyses involving the exploratory fibromyalgia cohort, were considered secondary or exploratory.

Group comparisons for continuous variables were performed using non-parametric Wilcoxon rank–sum (Mann–Whitney U) tests, reflecting the small sample sizes, potential non-normality of the metrics, and the use of matching primarily to reduce confounding rather than to define strictly paired hypotheses. Where appropriate, we report two-sided p-values with an alpha threshold of 0.05. Given the pilot nature of the study and the limited N, correction for multiple comparisons was applied to the primary endpoints only; secondary and exploratory analyses are interpreted cautiously, with emphasis on effect sizes and CIs rather than binary significance thresholds.

Effect sizes for group contrasts were summarised using Hedges’ g for approximately symmetric distributions and rank-biserial *r* for non-parametric tests. For each effect size, 95% CIs were estimated via bootstrap resampling (5000 resamples). For all main group contrasts, we report non-parametric test statistics alongside effect sizes and bootstrapped 95% CIs, either in the main text, in figure captions, or in Supplement. All inferential results reported in Figs. 1–3, Table 1 and Supplementary Tables S2–S7 and S11–S17 are based on the statistical procedures described in this section.

Analyses were conducted in Python 3.11.8 (Python Software Foundation) using NumPy 1.24.0^61^, SciPy 1.14.1^61^, statsmodels 0.13.5^62^ and pandas 1.5.3^63^. Visualisation used matplotlib 3.6.3^64^, seaborn 0.11.2^65^ and plotly 5.3.0^66^.

### Visualisation

All figures were generated in Python using matplotlib, seaborn and plotly as described above. Group-level summaries are plotted as mean ± SEM unless otherwise stated, with individual data points overlaid to show dispersion.

Key visualisations for primary and central secondary metrics (Z-AUC, LI, COI) include individual data points for patients and controls, sometimes with paired lines in summary plots, to facilitate visual assessment of dispersion and matching. Box-and-violin elements are used in some figures to convey distributional shape; point jitter is added where necessary to reduce overlap.

Z-profiles are presented as bar/line summaries in Fig. 2 with numeric values in Table S14. Colour palettes are held constant across figures to ensure that each diagnostic group is consistently represented (e.g. controls = grey, NT1 = gold, iRBD = blue, NREM parasomnia = magenta, fibromyalgia = red–orange).

In Fig. 2 (LI), one fibromyalgia subject showed an extreme LI value (≈ − 0.7); this point is displayed explicitly, and sensitivity analyses excluding this subject yielded similar group-level conclusions.

All main-text figures (Figs. 1–3) and supplementary figures (Supplementary Figs. S1–S3) were generated from the derived metrics using the visualisation approach outlined here.

### Multisite acquisition harmonisation and preprocessing

Patient EEGs and matched controls (MASS) were harmonised prior to Hori staging and metric derivation. All patient recordings were acquired at the KCL Sleep Disorders Centre using clinical PSG systems with standard scalp EEG, EOG and EMG channels; control recordings were obtained from the Montreal Archive of Sleep Studies (MASS), an open-access repository of laboratory-based PSG^52^.

Raw EEG signals were re-referenced to linked-mastoids, and filtered using identical digital bandpass and notch filters across sites (e.g. 0.3–35 Hz bandpass, 50 Hz notch). All data were downsampled to a common sampling rate of 250 Hz prior to visual inspection and Hori scoring. Acquisition parameters for patients and controls (MASS), including amplifier make/model, channels used for staging, reference montage, sampling rate and downsampling, anti-alias filtering, bandpass settings, notch filters, and preprocessing software, are summarised in Supplement Table S10.

No interpolation was performed on the specific EEG channels used for Hori staging (F3/F4, C3/C4, O1/O2); recordings with irreparable artefact on these channels during the onset interval were excluded from analysis. EOG and submentalis EMG channels were used to assist stage discrimination but were not included in quantitative metrics.

All patients and all controls were recorded at different respective sites; thus, site and case–control status are collinear and site could not be treated as an independent covariate in primary patient–control models. To mitigate site-related bias we restricted analyses to shared leads, applied identical referencing and filtering pipelines, used stage-wise Z-normalisation to matched controls, and emphasised within-subject or ordinal metrics (e.g. COI) that are comparatively insensitive to absolute amplitude scaling or minor montage differences. Additional patient-only within-site contrasts (see Cumulative ordering index section) provide a robustness check suggesting that the NT1 ordering effect is unlikely to be driven by site artefact.

Acquisition and harmonisation procedures described in this section are summarised in Supplementary Table S10 and underpin the within-site comparisons reported in Fig. 2 and Supplementary Table S7, as well as the AHI analyses in Supplementary Tables S12 and S13.

## Supplementary information

Supplementary Material

## Data Availability

The clinical polysomnography and EEG recordings analysed in this study contain sensitive health information and are subject to the data-protection policies of Guy’s and St Thomas’ NHS Foundation Trust and King’s College London. In accordance with the conditions of ethics approval (project number 12436) and UK data-protection legislation, raw recordings cannot be shared publicly. De-identified, derived data underlying the figures and tables (for example subject-level Hori stage durations and summary indices) may be made available from the corresponding author on reasonable request, subject to review and approval by the Trust’s Research and Development Office and the institutional Data Protection Officer. Control recordings were drawn from the Montreal Archive of Sleep Studies (MASS), which is publicly accessible as described in reference 52.

## Code availability

All analyses relied on standard open-source Python packages, as listed and cited in the Methods.
