# Supplementary material for: Disordered descent into sleep: microstructural divergence across arousal-linked conditions
Source: NPJ Biol Timing Sleep. 2026 Feb 26;3:8. doi: 10.1038/s44323-026-00070-8 (PMC12946162; doi:10.1038/s44323-026-00070-8)
Supplement: Supplementary file 1 — Supplementary Material [file 44323_2026_70_MOESM1_ESM.pdf]

Supplementary Material

Table S1. Demographic and Macrostructural Sleep Parameters

Group-level summary of demographic and polysomnographic metrics across diagnostic categories. This table presents key demographic and macrostructural sleep parameters for each patient group (Fibromyalgia Syndrome, Narcolepsy type 1, idiopathic REM sleep Behaviour Disorder [iRBD], and NREM parasomnia). Each column displays the mean ± standard deviation (SD) for the patient group, along with Hedges' g (standardised mean difference) and Mann–Whitney U test p-value calculated against their age and sex matched healthy control group. Sample sizes and percentage of female participants were: Narcolepsy type 1 (n = 12, 58% female), iRBD (n = 12, 42% female), NREM Parasomnia (n = 16, 50% female), Fibromyalgia Syndrome (n = 8, 100% female).

	**Fibromyalgia [Mean ± SD]**	**d**	**p**	**Narcolepsy**	**d**	**p**	**NREM Parasomnia**	**d**	**p**	**iRBD**	**d**	**p**
Age (years)	45.00 ± 8.12	-0.26	0.224	28.42 ± 11.07	-0.15	0.400	39.81 ± 7.30	-0.22	0.791	59.67 ± 7.69	0.01	0.884
TST (min)	301.88 ± 99.12	-1.36	0.028	393.70 ± 33.66	-0.88	0.046	382.43 ± 44.48	-1.14	0.005	374.62 ± 43.32	-1.22	0.0120
SOL(min)	35.30 ± 43.00	0.54	0.563	6.28 ± 4.67	-1.33	0.003	9.82 ± 7.41	-0.87	0.014	10.77 ± 6.15	-0.96	0.0376
SE (%)	65.08 ± 17.73	-1.33	0.028	84.57 ± 6.33	-0.58	0.184	85.31 ± 8.12	-0.13	0.880	79.37 ± 10.27	-0.38	0.7949
AI (ev/hr)	22.38 ± 7.63	2.37	0.000	16.32 ± 4.49	1.71	0.002	14.87 ± 6.67	0.58	0.105	22.73 ± 8.04	1.21	0.0193
WASO (min)	122.41 ± 64.32	1.09	0.049	66.04 ± 29.50	0.92	0.068	58.24 ± 42.75	0.14	0.895	87.96 ± 50.28	0.42	0.7508
AHI (ev/hr)	0.82 ± 1.56	-0.76	0.028	1.16 ± 1.43	-0.52	0.058	1.18 ± 1.94	-0.47	0.002	3.86 ± 3.60	0.76	0.1819
N1 (% TST)	13.57 ± 7.40	1.45	0.004	9.57 ± 5.52	0.62	0.174	8.75 ± 2.96	0.03	0.678	13.25 ± 5.93	0.19	0.9309
N2 (% TST)	41.81 ± 9.91	-0.25	0.573	43.14 ± 7.85	-0.67	0.140	47.91 ± 8.57	-0.06	1.0	43.57 ± 9.42	-0.45	0.4024
N3 (% TST)	24.98 ± 7.92	-0.17	0.878	25.90 ± 5.95	0.84	0.040	21.52 ± 8.52	0.14	0.806	23.69 ± 5.91	0.84	0.0404
REM (% TST)	19.65 ± 8.35	-0.64	0.194	21.38 ± 5.18	-0.45	0.340	21.83 ± 4.83	-0.13	0.806	19.50 ± 5.81	-0.39	0.3262
SL to N2 (min)	42.24 ± 42.40	0.65	0.318	10.41 ± 7.26	-1.22	0.007	14.04 ± 8.36	-0.84	0.021	15.06 ± 7.71	-0.92	0.0461
SL to N3 (min)	80.80 ± 62.45	1.05	0.160	20.32 ± 10.59	-1.22	0.010	28.17 ± 13.14	-0.69	0.047	24.97 ± 11.62	-1.11	0.0192
SL to REM (min)	–	–	–	90.04 ± 32.90	0.4	0.236	86.31 ± 23.64	-0.16	0.806	110.88 ± 77.06	0.67	0.1260

Abbreviations: TST = Total Sleep Time; WASO = Wake After Sleep Onset; SE = Sleep Efficiency; SL = Sleep Latency; N1/2/3% = Percentage of TST in sleep stages N1/2/3; REM% = Percentage of TST in REM sleep; AHI = Apnoea–Hypopnea Index (events/hour); AI = Arousal Index (arousals/hour).

Table S2. Group-Wise Laterality Indices and Effect Sizes

Group	Mean	Δ LI	Hedges g	p
Fibromyalgia	-0.143	-0.129	-0.635	0.701
Narcolepsy type 1	0.008	0.009	0.216	0.111
NREM parasomnia	-0.068	-0.042	-0.280	0.724
iRBD	0.020	0.064	0.637	0.073

***Note**: Positive values denote right-hemispheric predominance. Values are means; effect sizes are Hedges' g. p values are from Mann–Whitney U (two-sided).*

Z-Normalised Stage-wise Sleep Onset Analysis

To characterise microstructural disruption in the sleep onset process, we derived stage-specific durations for Hori stages 4 through 10. This method allowed precise quantification of the dwell time within each Hori substage, consistent with the physiological definition of microstructural transitions during sleep onset.

Stage-wise durations were then Z-score normalised relative to the mean and standard deviation of matched controls at each corresponding Hori stage. This approach preserves both anatomical and temporal resolution while enabling cross-subject comparability independent of absolute sleep latency. By standardising per stage, we avoided conflating accelerated transitions in one substage with prolongation in another, thus isolating the specific phases of sleep onset most affected by each clinical condition.

Table S3. Stage-wise Z-Normalised Sleep Onset Deviations.

Mean Z-score deviations for Hori stages 4–10 across diagnostic groups. For each stage, durations were standardised relative to the matched control group's mean and standard deviation. Values reflect the direction and magnitude of deviation from normative sleep onset timing. Mann–Whitney U statistics and Hedges' g effect sizes quantify the difference between each patient group and its matched controls at each stage.

Disorder	H4	H5	H6	H7	H8	H9	H10
Fibromyalgia	3.51	5.80	-0.41	0.43	-0.06	1.59	1.10
Narcolepsy type 1	-0.34	-0.36	-0.25	0.61	-0.53	0.62	-0.40
NREM Parasomnia	-0.05	0.20	-0.21	-0.42	-0.15	0.12	-0.15
iRBD	0.29	0.96	-0.28	-0.10	-0.32	0.07	0.05

Entropy and Euclidean Deviation Metrics

To characterise temporal disorder in the sleep onset microstructure, we computed two complementary measures across Hori stages 4 through 10: Shannon entropy and Euclidean distance. For each subject, Hori stage durations were converted into relative proportions of the total sleep onset period, from which Shannon entropy (H) was calculated:

$H = -\Sigma\ p_i \log_2(p_i)$, where p_i is the proportion of time spent in stage i (i = 4–10).

Higher entropy values indicate more uniform dwell time distributions, while lower entropy reflects stage dominance or compression. Euclidean deviation (ED) was computed for each subject's Hori stage duration vector relative to their matched control group mean vector:

$ED = \sqrt{\Sigma(x_i - \mu_i)^2}$, where x_i is the subject's stage duration and μ_i is the control group mean for stage i.

Larger distances reflect greater deviation from normative temporal structure. Group-level comparisons were conducted using Mann–Whitney U tests and Cohen's d effect sizes.

Table S4. Stage- Occupancy Entropy.

Stage occupancy entropy quantifies the distribution of dwell times across H4–H10.

Clinical Group	Mean	SEM	Hedges g	M–W U	p-value
Fibromyalgia	1.631	0.178	-0.566	21	0.2786
Narcolepsy Type 1	1.836	0.128	0.5	88	0.3708
NREM Parasomnia	1.65	0.163	0.003	132	0.8951
iRBD	1.669	0.2	0.198	79	0.7075

Note: *Shannon entropy of H4–H10 dwell-time proportions (bits). Mann–Whitney U vs matched controls; effect size is Hedges' g.*

Table S5. Euclidean Distance from Control Onset Profiles.

ED is the Euclidean distance between each subject's H4–H10 vector and the matched-control mean vector.

Clinical Group	Mean ED	SEM	Hedges g	M–W U	p-value
Fibromyalgia	8.874	2.232	1.17	49	0.083
Narcolepsy type 1	4.033	0.373	-0.629	53	0.2855
NREM Parasomnia	3.515	0.338	-0.535	97	0.2503
iRBD	4.492	0.597	-0.417	62	0.5834

Abbreviations: SEM = standard error of the mean; M–W U = Mann–Whitney U test; iRBD = idiopathic REM sleep behaviour disorder; NREM = NREM sleep.

Table S6. Disorder-Specific Correlation Divergence from Matched Controls.

This table lists a bootstrapped comparison of correlation strengths (Δr) between microstructural and macrostructural sleep parameters in clinical populations versus their matched control groups.

For each significant disorder-specific correlation (|r| ≥ 0.5), the corresponding correlation in controls was computed and subtracted to yield Δr = r_patient − r_control. Bootstrapped 95% confidence intervals (CI) for the difference were calculated using 1000 iterations of paired resampling. Microstructural metrics included: (1) stage occupancy entropy (H4–H10), reflecting distributional uniformity of dwell times; (2) Euclidean distance from the group-matched control trajectory; (3) Laterality Index (LI), defined as LI = (R − L)/(R + L). *For the primary analyses, Δr was considered statistically significant if its bootstrapped 95% confidence interval excluded zero. However, all micro–macro correlation analyses reported here, including those in narcolepsy type 1, were pre-specified as exploratory in a small sample; accordingly, we cautiously treat these Δr estimates as descriptive and do not regard confidence intervals excluding zero as confirmatory evidence. The pre-specified pairing in NT1 was entropy (stage-occupancy) with total sleep time (TST).* Presented correlations observed in narcolepsy type 1 patients only, may potentially represent disorder-specific physiological reorganisation.

Clinical Group	**Microstructural Metric**	**Macrostructural Feature**	**Δr**	**95% CI for Δr**	**Interpretation**
Narcolepsy type 1	Entropy	REM %	0.72	[0.09, 1.36]	Only descriptive, not significant. Greater entropy associated with increased REM sleep proportion
Narcolepsy type 1	Entropy	N1 %	-0.69	[-1.13, -0.12]	Only descriptive, not significant. Greater entropy associated with decreased N1 sleep proportion (light sleep)
Narcolepsy type 1	Entropy	TST	0.725	[0.027, 1.262]	Greater entropy associated with increased total sleep time

Table S7. Cumulative Ordering Index (COI) by Group.

Group means (±SEM) for COI inversion ratio and inversion magnitude (seconds). Mann–Whitney U tests compare patients with matched controls; rank biserial r shown as effect size. NT1 shows higher inversion ratio and magnitude than controls (p ≈ 0.036–0.037); other groups are near zero or trend level only.

Group	COI (ratio)	U (ratio)	p (ratio)	r (ratio)	COI inversion magnitude	U	p	r
Fibromyalgia (patients)	0.000 ± 0.000	28	0.3816	0.125	0.000 ± 0.000	28	0.3816	0.125
NREM Parasomnia	0.000 ± 0.000	120	0.3485	0.062	0.000 ± 0.000	120	0.3485	0.062
Narcolepsy Type 1	0.048 ± 0.020	96	0.0357	-0.333	1.686 ± 0.855	96	0.0367	-0.333
iRBD	0.024 ± 0.016	84	0.1658	-0.167	1.602 ± 1.598	84	0.1662	-0.167

Note: COI ratio is the fraction of adjacent cumulative substage pairs where time(1→K+1) < time(1→K). Inversion magnitude is the sum of negative deltas (seconds). Mann–Whitney U compares patients vs controls within each group; r is rank-biserial effect size.

Derived measures. Substage timing markers available across the onset window were used to compute dwell times for H4–H10 and hemispheric totals. Stage occupancy entropy was computed from H4–H10 dwell time proportions. COI was defined as the fraction of adjacent cumulative substage pairs where time(1→K+1) < time(1→K); inversion magnitude is the sum of negative deltas (s).

Arousal Regulation Dynamics in Sleep and Pain Disorders

Recent theoretical models in systems neuroscience, such as that proposed by LeDuke et al. (2023), have delineated the organisation of psychiatric traits along dynamic attractor landscapes governed by top-down and bottom-up circuit interactions[42]. This framework offers a compelling scaffold to interpret arousal dysregulation as a function of neural stability, switching capacity, and plasticity timescales. Here, we extend this model into the domain of neurophysiologically measurable sleep and pain-related disorders. By mapping disorders such as narcolepsy, fibromyalgia, parasomnia, and idiopathic REM Behaviour Disorder (iRBD) onto discrete attractor regimes, we highlight the translational potential of EEG-derived metrics, such as entropy, Z-transformed area under the curve (Z-AUC), and interhemispheric asymmetry, as tools to quantify latent circuit dynamics. This synthesis integrates Buzsáki's conception of state-space architecture with clinical neurobiology[68,69], proposing that the degree and direction of arousal-state fragmentation can serve as a mechanistic bridge between psychiatric phenotypes and observable electrophysiological microstructure.

Table S8. Mapping Clinical Sleep Disorders onto Theoretical Attractor State Profiles[42].

Clinical Group	Mapped Attractor Profile	Mapped Circuit Type	Mechanistic Features	Representative EEG Signatures	Supporting Features from Hori Metrics
Narcolepsy Type 1	Shallow, Unstable Attractors	Comorbid (Fast Switching)	Hyper-reactivity, impaired boundary gating	High entropy, unstable CAP/Hori transitions	Compressed onset, high entropy, low Z-AUC, rapid state switching
Fibromyalgia Syndrome	Fragmented Attractors	Anxious	Hypervigilance, excessive bottom-up salience	Alpha intrusion, elevated micro-arousals	Prolonged early stages, high Z-AUC, elevated ED, increased hemispheric asymmetry
iRBD	Multistable / Over-constrained Attractors	Depressed	Rigid cortical control, hypodopaminergic slowing	Reduced reactivity, blunted REM microstructure	Moderate onset disruption, REM-related dyscontrol, asymmetric LI
NREM Parasomnia	Shallow or Fragmented Attractors	Comorbid (Slow Switching)	Instability + weak integration of top-down inhibition	State fragmentation, lateralised delta asymmetry	Mild onset prolongation, near-normative entropy, partial lateralisation

Abbreviations. Hori = Hori microstaging system; ED = Euclidean distance; Z-AUC = cumulative Z-score deviation across stages; LI = Laterality Index; iRBD = idiopathic REM sleep behaviour disorder.

Legend: This table presents a hypothesis-driven mapping of sleep disorder phenotypes onto the attractor state framework originally proposed by LeDuke et al. (2023)[42]. Attractor depth and structure were inferred from temporal microstructural dynamics during sleep onset,

including timing consistency, transition irregularity, and hemispheric asymmetry. These alignments are intended to offer a theoretical systems-level interpretation of the physiological patterns observed in each disorder.

Table S9. Neuropsychiatric Comorbidity Across Sleep and Pain-Related Disorders.

Clinical Group	Depression	Anxiety	Other Psychiatric
Narcolepsy	32–35% (BOND study[36])	25.7–27.5%	↑ ADHD[45], psychotic traits
Insomnia Disorder	~40% with psychiatric comorbidity[70]	Frequent comorbidity	Predictive of later major depression[71]
Fibromyalgia	Up to 30% (3–5× general population)[37]	~30–33% PTSD[38]; anxiety elevated[44]	Mood disorders, somatoform symptoms
Idiopathic REM Sleep Behavior Disorder (iRBD)	Frequently comorbid[43]; exact % unclear	Common but under-quantified[39]	Prodrome to synucleinopathies
NREM Parasomnia	Linked to trauma and mood instability[72]	Often comorbid with PTSD and anxiety	Linked to dissociative symptoms, trauma[40]

Legend: This table summarises published estimates of psychiatric comorbidity across sleep and chronic pain conditions, based on ICD and ICSD diagnostic categories.

Abbreviations: iRBD – Idiopathic REM Sleep Behavior Disorder; PTSD – Post-Traumatic Stress Disorder; ADHD – Attention Deficit Hyperactivity Disorder.

Table S10. Acquisition and preprocessing parameters for patients (KHP) and controls (MASS).

Parameter	Patients	MASS controls (Montreal Archive of Sleep Studies)
Recording environment	Hospital-based clinical sleep laboratory (standard overnight diagnostic PSG).	Hospital-based laboratory PSG recordings pooled from several research protocols.
PSG system / amplifier	Standard clinical PSG systems used at the Sleep Disorders Centre (GSTT); specific amplifier models varied across years but were not altered for this analysis.	Standard clinical PSG systems used in the contributing Montreal sleep laboratories; specific amplifier models are not specified in the public MASS documentation.
EEG channels used for Hori staging	F3, F4, C3, C4, O1, O2 (10–20 system), plus bilateral EOG and submentalis EMG.	Same subset extracted from the MASS montage: F3, F4, C3, C4, O1, O2 (10–20

		system), plus bilateral EOG and submentalis EMG.
Original EEG montage	Full clinical PSG montage including standard 10–20 EEG channels, bilateral EOG, chin EMG, ECG and respiratory sensors, as previously published[73].	Full PSG montage with 4–20 EEG channels placed according to the 10–20 system, plus standard EOG, EMG, ECG and respiratory signals.
Reference scheme in analysis	All EEG channels re-referenced offline to averaged mastoids (M1/M2) before Hori staging and metric derivation.	All EEG channels re-referenced offline to averaged mastoids (M1/M2).
Native EEG sampling rate	Clinical acquisition at ≥256 Hz; all EEG channels resampled to 256 Hz for harmonised analysis.	EEG channels acquired at 256 Hz in all MASS recordings.
Sampling rate used for analysis	256 Hz for all EEG channels after resampling with an anti-alias filter.	256 Hz (native MASS sampling rate), processed with the same filtering and preprocessing steps as KCL data.
Bandpass filter (analysis)	0.3–35 Hz zero-phase digital bandpass filter applied identically to all EEG channels before staging and metric derivation.	Same 0.3–35 Hz zero-phase digital bandpass filter applied to MASS EEG channels.
Notch filter (line noise)	50 Hz notch filter (UK mains frequency) applied to EEG channels using a single implementation across all recordings.	60 Hz notch filter (North American mains frequency) applied with the same implementation; only the notch centre frequency differed from KCL.
Downsampling / anti-aliasing	When native sampling exceeded 256 Hz, EEG was low-pass anti-alias filtered and resampled to 256 Hz using the same Python preprocessing pipeline.	MASS EEG was acquired at 256 Hz and processed with the same filters; no additional downsampling beyond this common 256 Hz analysis rate.
Preprocessing software	Custom Python-based pipeline (Python 3.11) using the standard scientific Python stack for re-referencing, filtering, downsampling, and artefact screening.	The same Python-based preprocessing pipeline and scripts applied to MASS recordings, ensuring identical re-referencing, filtering, downsampling, and artefact criteria.
Artefact handling for staging channels	No interpolation performed on F3, F4, C3, C4, O1, O2.	Same criterion: MASS recordings with irreparable

	Recordings with irreparable artefact on any of these channels in the sleep-onset interval were excluded from analysis.	artefact on F3, F4, C3, C4, O1 or O2 during the onset interval were excluded rather than interpolated.

Table S11. Age matching of patient–control pairs across diagnostic cohorts.

Group	N pairs	Patient age (years) mean ± SD	Control age (years) mean ± SD	ΔAge (patient – control) mean ± SD	ΔAge range (years)	|ΔAge| (years) mean ± SD	|ΔAge| range (years)
Fibromyalgia	8	45.0 ± 8.1	47.6 ± 11.9	-2.6 ± 16.2	-25.0 to 22.0	12.6 ± 9.3	1.0 to 25.0
Narcolepsy type 1	12	28.4 ± 11.1	30.0 ± 10.5	-1.6 ± 10.8	-26.0 to 18.0	7.8 ± 7.3	1.0 to 26.0
iRBD	12	59.7 ± 7.7	59.7 ± 6.0	0.0 ± 12.5	-17.0 to 20.0	11.0 ± 5.0	4.0 to 20.0
NREM parasomnia	16	39.8 ± 7.3	42.2 ± 13.5	-2.4 ± 15.3	-23.0 to 18.0	13.4 ± 7.1	0.0 to 23.0
All cohorts	48	42.8 ± 14.1	44.4 ± 15.1	-1.6 ± 13.4	-26.0 to 22.0	11.2 ± 7.2	0.0 to 26.0

Age matching statistics for patient–control pairs in each diagnostic cohort and for the full sample. Values are mean ± SD (years) unless otherwise indicated. ΔAge denotes the age difference between each patient and their matched control (patient minus control); |ΔAge| denotes the absolute age difference.

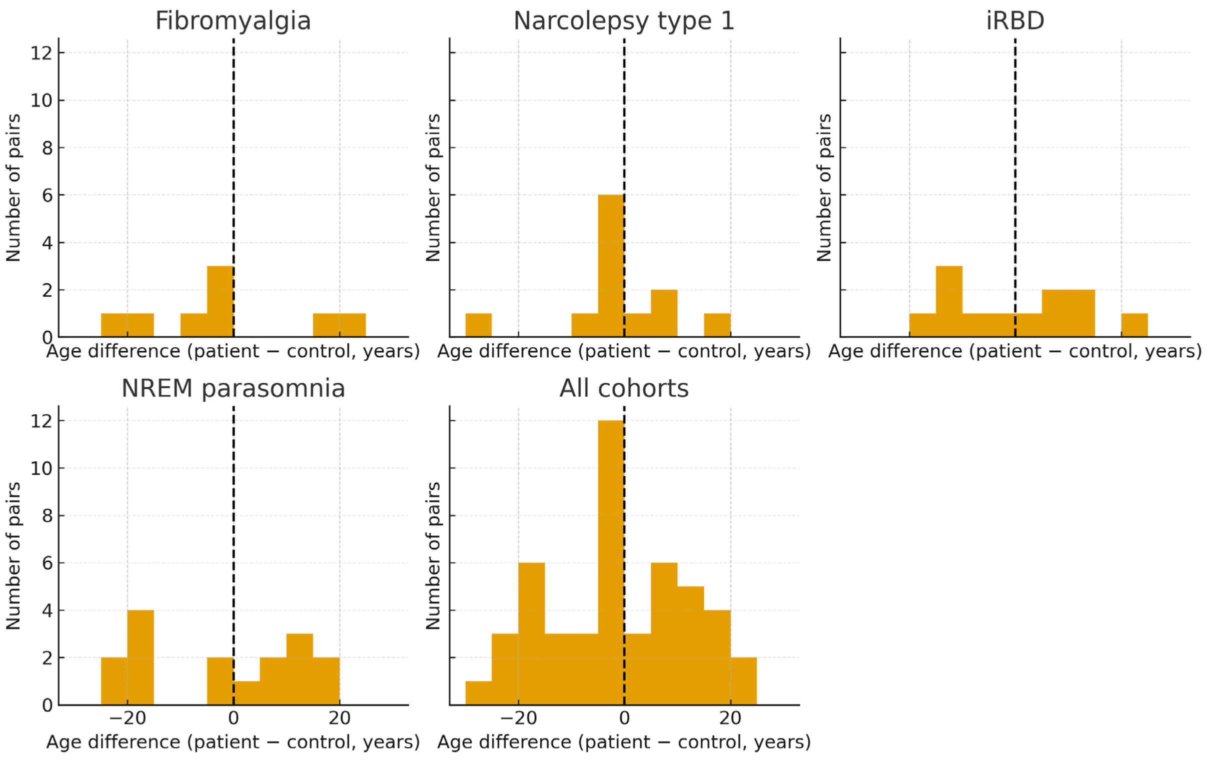

Figure S1. Distribution of patient–control age differences across diagnostic cohorts. Histograms illustrating the distribution of age differences for each diagnostic cohort and for all patient–control pairs combined (age difference = patient age − matched control age, in years). Each panel displays counts of pairs within 5-year bins, with a vertical dashed line at zero indicating perfect age matching. Across cohorts, age differences are approximately symmetric around zero and predominantly clustered within a moderate window, with a small number of more widely separated pairs captured in the tails. These distributions visually confirm that patient and control ages are broadly comparable within cohorts and support the validity of the age-matching procedure summarised in Table S11.

Table S12. AHI distributions.

AHI (events/hour) was extracted from the intragroup patient files and the corresponding control files for each disorder. For NT1 and matched controls, all subjects had AHI < 5 events/hour. For iRBD and NREM parasomnia patients, a small number of individuals exhibited AHI ≥ 5, but none exceeded AHI ≥ 15, consistent with the study's exclusion criteria.

Group	N patients	Patient AHI mean ± SD	N controls	Control AHI mean ± SD	Patients with AHI ≥ 5 (n, %)	Patients with AHI ≥ 10 (n, %)
Fibromyalgia	8	0.8 ± 1.6	8	1.8 ± 0.7	0 (0.0%)	0 (0.0%)
Narcolepsy	12	1.2 ± 1.4	12	1.8 ± 0.8	0 (0.0%)	0 (0.0%)
iRBD	12	3.9 ± 3.6	12	1.9 ± 0.5	3 (25.0%)	1 (8.3%)
NREM parasomnia	16	1.2 ± 1.9	16	1.9 ± 0.7	1 (6.2%)	0 (0.0%)

Table S13. AHI distributions and NT1 primary endpoint sensitivity to AHI cut-offs.

The upper panel summarises apnoea–hypopnoea index (AHI, events/hour) for each diagnostic group and matched controls, reporting mean ± SD AHI and the number and percentage of patients with AHI ≥ 5 and ≥ 10. The lower panel provides sensitivity analyses for the NT1 primary endpoints (Z-AUC and COI) under three AHI thresholds (no restriction, AHI < 10, AHI < 5), listing sample sizes, mean ± SD Z-AUC and COI in patients and controls, and Mann–Whitney p-values. Because all NT1 patients and controls in this sample have AHI < 5, Z-AUC and COI values are identical across thresholds, indicating that the NT1 findings are not driven by occult obstructive sleep apnoea.

AHI cutoff	N patients	N controls	Z-AUC patients mean ± SD	Z-AUC controls mean ± SD	p (Z-AUC, MWU)	COI patients mean ± SD	COI controls mean ± SD	p (COI, MWU)	Note
None	12	12	-0.7 ± 5.3	-0.0 ± 2.5	0.112	0.048 ± 0.070	0.0 ± 0.0	0.036	All AHI < cutoff
AHI < 10	12	12	-0.7 ± 5.3	-0.0 ± 2.5	0.112	0.048 ± 0.070	0.0 ± 0.0	0.036	All AHI < cutoff
AHI < 5	12	12	-0.7 ± 5.3	-0.0 ± 2.5	0.112	0.048 ± 0.070	0.0 ± 0.0	0.036	All AHI < cutoff

Table S14. H4–H10 control baselines and raw dwell times for patients and controls.

Panel A: Control baselines for H4–H10 dwell times. For each diagnostic-specific control group, mean ± SEM dwell times (seconds) for Hori substages H4–H10 are shown together with the number of controls (N). These values were used as the per-stage baselines for Z-normalisation.

Group	Hori substage	N controls	Mean dwell time (s)	SEM (s)
Fibromyalgia	H4	8	0.917	0.446
Fibromyalgia	H5	8	2.917	1.144
Fibromyalgia	H6	8	0.573	0.191
Fibromyalgia	H7	8	0.469	0.229
Fibromyalgia	H8	8	0.495	0.141
Fibromyalgia	H9	8	0.552	0.147
Fibromyalgia	H10	8	0.552	0.147
Narcolepsy	H4	12	2.819	1.618
Narcolepsy	H5	12	1.191	0.546
Narcolepsy	H6	12	0.427	0.115
Narcolepsy	H7	12	1.038	0.382
Narcolepsy	H8	12	0.694	0.290
Narcolepsy	H9	12	1.788	0.795
Narcolepsy	H10	12	1.788	0.795
NREM Parasomnia	H4	16	1.198	0.592
NREM Parasomnia	H5	16	2.047	0.755
NREM Parasomnia	H6	16	0.760	0.233
NREM Parasomnia	H7	16	0.914	0.374
NREM Parasomnia	H8	16	0.891	0.251
NREM Parasomnia	H9	16	1.221	0.600
NREM Parasomnia	H10	16	1.221	0.600
iRBD	H4	12	0.889	0.363
iRBD	H5	12	3.264	1.929
iRBD	H6	12	0.976	0.484
iRBD	H7	12	1.635	0.870
iRBD	H8	12	1.059	0.470
iRBD	H9	12	0.451	0.221
iRBD	H10	12	0.451	0.221

Panel B: Raw H4–H10 dwell times and Z-AUC in patients and controls. For each diagnostic group and role (patient, control), this panel reports the mean ± SEM dwell time (seconds) in each Hori substage H4–H10 and the mean ± SEM cumulative Z-AUC. These raw values clarify how absolute dwell times give rise to the Z-normalised microstructural profiles in Figure 1 and underpin the groupwise Z-AUC comparisons shown in Figure 2.

Group	Role	N	H4 (mean ± SEM, s)	H5	H6	H7	H8	H9	H10	Z-AUC (mean ± SEM)
Fibromyalgia	Patient	8	8.23 ± 2.71	1.60 ± 0.51	0.81 ± 0.44	0.43 ± 0.20	1.13 ± 0.36	1.01 ± 0.33	1.01 ± 0.33	9.55 ± 2.69
Fibromyalgia	Control	8	0.92 ± 0.45	2.92 ± 1.14	0.57 ± 0.19	0.47 ± 0.23	0.49 ± 0.14	0.55 ± 0.15	0.55 ± 0.15	0.00 ± 1.02
Narcolepsy	Patient	12	0.78 ± 0.24	0.72 ± 0.19	0.67 ± 0.34	0.34 ± 0.19	1.32 ± 0.58	0.69 ± 0.41	0.69 ± 0.41	-0.71 ± 1.54
Narcolepsy	Control	12	2.82 ± 1.62	1.19 ± 0.55	0.43 ± 0.12	1.04 ± 0.38	0.69 ± 0.29	1.79 ± 0.80	1.79 ± 0.80	-0.00 ± 0.73
NREM Parasomnia	Patient	16	1.67 ± 0.52	1.41 ± 0.46	0.37 ± 0.11	0.69 ± 0.22	1.01 ± 0.26	0.85 ± 0.36	0.85 ± 0.36	-0.77 ± 0.53
NREM Parasomnia	Control	16	1.20 ± 0.59	2.05 ± 0.76	0.76 ± 0.23	0.91 ± 0.37	0.89 ± 0.25	1.22 ± 0.60	1.22 ± 0.60	0.00 ± 0.70
iRBD	Patient	12	2.09 ± 0.81	1.40 ± 0.80	0.81 ± 0.28	0.68 ± 0.28	1.18 ± 0.35	0.49 ± 0.21	0.49 ± 0.21	0.44 ± 1.02
iRBD	Control	12	0.89 ± 0.36	3.26 ± 1.93	0.98 ± 0.48	1.64 ± 0.87	1.06 ± 0.47	0.45 ± 0.22	0.45 ± 0.22	0.00 ± 0.67

Hori substage codes: H4–H10 denote the microstructural stages used for Z-normalised onset analyses. Dwell times are expressed in seconds and derived from Hori 4-s epoch counts over the onset window. Z-AUC is the sum of per-stage Z-scores across H4–H10 relative to the diagnostic-specific control baselines in Panel A.

Table S15. Inter-rater Hori Metrics.

Inter-rater reliability was assessed on a double-rated subset of 900 onset epochs (4-s mini-epochs across all groups). The upper panel shows the full 7 × 7 confusion matrix for H4–H10 (rows = Rater 1, columns = Rater 2), with diagonal entries indicating exact agreement and off-diagonal entries reflecting disagreements, predominantly between adjacent substages. The lower panel reports, for each substage H4–H10, the number of epochs in which at least one rater assigned that stage, the overall agreement across all epochs, the agreement conditional on that stage being used by either rater. Overall multi-category Cohen's κ for H4–H10 across all 900 epochs was 0.745, with an observed agreement proportion (P_o) of 0.791.

Panel A: Confusion matrix.

Rater 1 \ Rater 2	H4	H5	H6	H7	H8	H9	H10
H4	38	7	0	1	0	0	0
H5	9	71	7	1	0	0	2
H6	0	15	137	17	0	1	2
H7	2	1	22	187	22	1	6
H8	3	1	1	14	149	20	1
H9	1	1	0	1	15	96	9
H10	0	0	1	0	0	4	34

Diagonal entries indicate exact agreement on the Hori substage; off-diagonal entries reflect disagreements, which are predominantly between adjacent substages (e.g. H6 vs H7, H7 vs H8).

Panel B. Per-stage agreement. For each Hori substage H4–H10, we computed the number of epochs in which at least one rater assigned that stage, the overall agreement across all epochs, the conditional agreement given that at least one rater used that stage.

Stage	n epochs with stage (any rater)	Agreement overall (%)	Agreement given stage (%)
H4	61	97.4	62.3
H5	115	95.1	61.7
H6	203	92.7	67.5
H7	275	90.2	68.0
H8	226	91.4	65.9
H9	149	94.1	64.4
H10	59	97.2	57.6

Conditional agreement given that at least one rater used a stage ranges from approximately 58–68% across H4–H10.

Table S16. Female-only fibromyalgia sensitivity analysis (Z-AUC, entropy, ED, LI, COI).

This table summarises the female-only fibromyalgia sensitivity analysis. All eight fibromyalgia patients and their eight matched controls are women. For Z-AUC, entropy (bits), Euclidean deviation (ED), the hemispheric Laterality Index, and COI, the table reports mean ± SD values in patients and controls, Hedges' g with 95% bootstrap confidence intervals (5,000 resamples), and Mann–Whitney p-values. As in the main analysis, Z-AUC shows a large positive effect in fibromyalgia relative to female controls, whereas differences in entropy, ED, LI and COI are smaller with confidence intervals that include zero and are therefore treated as exploratory.

**Metric**	**Patients (n=8) mean ± SD**	**Controls (n=8) mean ± SD**	**Hedges g**	**95% CI for g**	**p (Mann–Whitney)**	**Interpretive note**
Z-AUC	9.55 ± 7.61	0.00 ± 2.88	1.57	[0.76, 3.67]	0.015	Patients > controls (CI excludes 0).
Entropy (bits)	1.63 ± 0.50	1.92 ± 0.47	-0.57	[-1.77, 0.35]	0.279	Difference is exploratory (CI includes 0).
Euclidean deviation (ED)	8.87 ± 6.31	3.29 ± 0.96	1.17	[0.48, 2.57]	0.083	Patients > controls (CI excludes 0).
Laterality Index	-0.14 ± 0.27	-0.01 ± 0.04	-0.63	[-1.35, 0.67]	0.701	Difference is exploratory (CI includes 0).
COI	0.00 ± 0.00	0.02 ± 0.06	-0.47	[-0.97, 0.00]	0.382	Difference is exploratory (CI includes 0).

As expected from the main analysis, Z-AUC shows a large positive effect (Hedges' g ≈ 1.6, 95% CI excluding zero), indicating prolonged, divergent onset in the exploratory fibromyalgia cohort relative to female controls. Differences in entropy, ED, LI and COI are smaller and have confidence intervals that include zero, and are therefore treated as exploratory and hypothesis-generating.

COI Distribution and Null Simulations

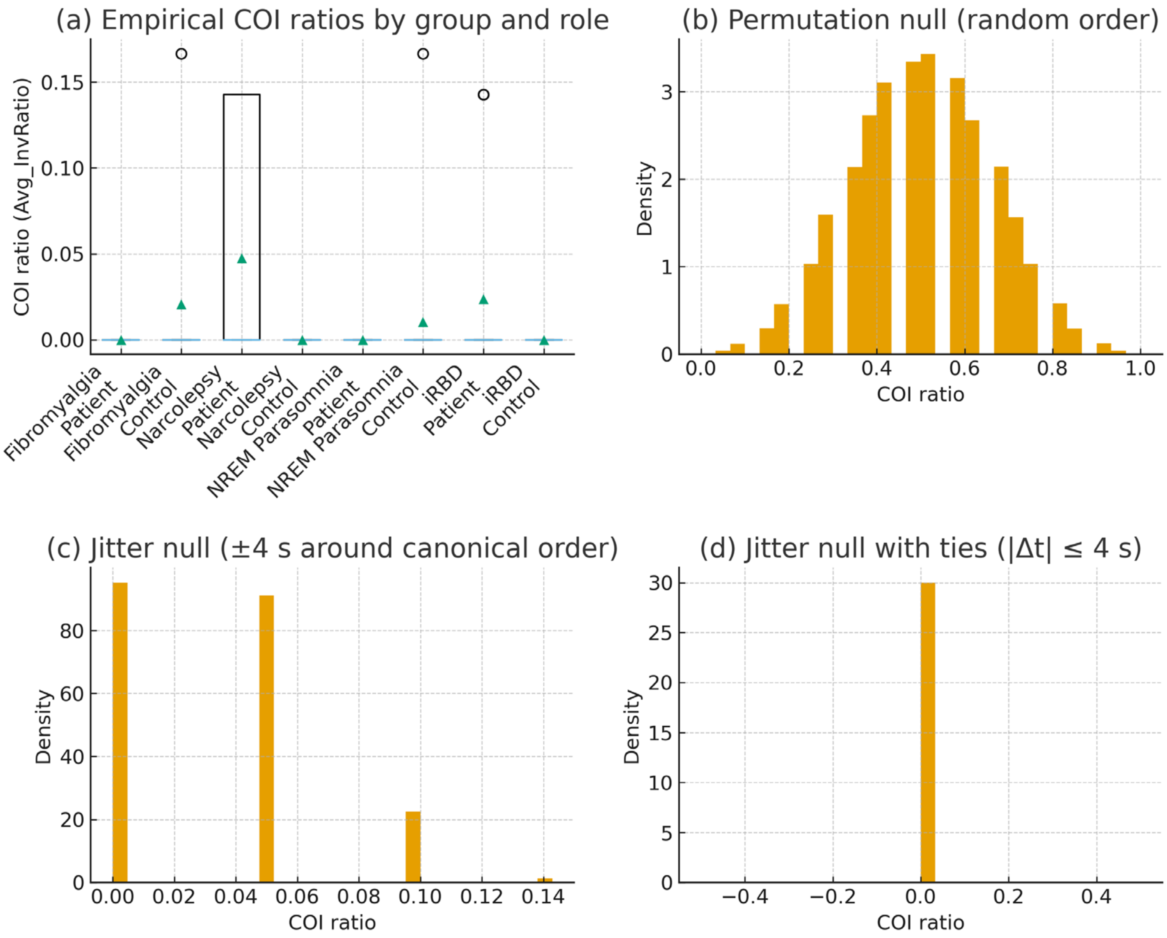

Figure S2. COI distributions across groups and simulation-based null models. Panel (a) shows empirical COI ratios and total inversion magnitudes for patients and controls in each diagnostic group, highlighting that controls are clustered near zero while NT1 patients alone show clearly elevated COI. Panel (b) depicts the permutation-based null distribution of COI ratios and magnitudes for H4–H10 when substage first-arrival orders are randomly permuted, with empirical group means overlaid. Panel (c) shows the jitter null, in which canonical arrival times are perturbed by ± one 4-s epoch, and panel (d) the jitter-plus-ties null in which near-simultaneous arrivals (± 4 s) are treated as ties. These panels illustrate that NT1 COI values lie far below permutation-null expectations, above the jitter-null mean, and remain elevated under a conservative ties rule, whereas control COI values remain at or near floor under all nulls.

Empirical COI distributions

Group	Role	n	COI ratio mean ± SD	COI magnitude mean ± SD
Fibromyalgia	Control	8	0.021 ± 0.059	0.062 ± 0.177
Fibromyalgia	Patient	8	0.000 ± 0.000	0.000 ± 0.000
NREM Parasomnia	Control	16	0.010 ± 0.042	0.031 ± 0.125
NREM Parasomnia	Patient	16	0.000 ± 0.000	0.000 ± 0.000
Narcolepsy	Control	12	0.000 ± 0.000	0.000 ± 0.000
Narcolepsy	Patient	12	0.048 ± 0.070	1.686 ± 2.961
iRBD	Control	12	0.000 ± 0.000	0.000 ± 0.000
iRBD	Patient	12	0.024 ± 0.056	1.602 ± 5.534

As expected, controls in all groups show COI ratios and magnitudes clustered very close to zero, indicating near-canonical Hori ordering. NT1 patients are the only group with a clearly elevated mean COI ratio (~0.048) and non-zero inversion magnitude, consistent with the main analysis. For these null simulations we used an all-pairs generalisation of the COI metric (inversions computed over all ordered substage pairs) for analytic convenience; the empirical COI used in the main analyses is defined over adjacent canonical pairs as in Methods "Cumulative Ordering Index (COI)" section, and the two measures are tightly correlated and yield the same qualitative group pattern.

Permutation null model

To characterise the expected distribution of COI under random ordering, we simulated synthetic onset trajectories for a canonical set of seven Hori substages (H4–H10) by randomly permuting the first-arrival order of these stages. For each simulation, we computed inversion counts over all ordered pairs of substages, the corresponding inversion ratio (inversions / total pairs), and the total inversion magnitude (sum of time-order violations). This procedure was repeated for 50,000 simulated subjects.

Null model	Mean COI ratio	SD COI ratio	95th percentile	99th percentile	Max
Permutation (ratio)	0.502	0.158	0.762	0.857	1.000
Permutation (magnitude)	28.12	11.43	47.00	52.00	56.00

Under this permutation null, the expected COI ratio is approximately 0.50 with substantial variability (SD ≈ 0.16), and values above ~0.76 and ~0.86 correspond to the 95th and 99th percentiles, respectively. The empirical COI ratios observed in controls and in most patient groups are far below this range, while NT1 patient ratios (~0.05) sit in the extreme lower tail of the permutation null, reflecting structured, low-inversion trajectories rather than randomised order.

Jitter null model (±4 s)

To approximate the impact of modest scorer or timing jitter, we constructed a second null model in which each canonical stage time was jittered by ± one 4-s mini-epoch. Canonical first-arrival times were set to 0, 1, …, 6 for H4–H10, and at each simulation a discrete jitter in {−1, 0, +1} epochs was added independently to each stage. COI inversion counts, ratios, and magnitudes were then recomputed over all ordered substage pairs for 50,000 simulated subjects.

Null model	Mean COI ratio	SD COI ratio	95th percentile	99th percentile	Max
Jitter ±4 s (ratio)	0.032	0.033	0.095	0.095	0.143
Jitter ±4 s (magnitude)	0.66	0.69	2.00	2.00	3.00

Under the jitter null, the expected COI ratio is close to zero (mean ≈ 0.032, SD ≈ 0.033), with a 95th percentile around 0.095. This reflects the fact that small, symmetric jitter around the canonical order produces relatively few inversions. The empirical NT1 COI ratios (~0.05) lie above the jitter null mean and within its upper tail, whereas control COI ratios remain clustered very close to zero.

Jitter null with tolerant "ties" rule (±4 s)

Finally, to emulate a more conservative scoring rule in which near-simultaneous arrivals are treated as ties, we repeated the jitter simulations but ignored any pair of substages whose jittered times differed by at most one 4-s mini-epoch ($|\Delta t| \le 1$). Such pairs neither contributed to inversion counts nor to the denominator of the COI ratio.

Null model	Mean COI ratio	SD COI ratio	95th percentile	99th percentile	Max
Jitter ±4 s + ties (ratio)	0.000	0.000	0.000	0.000	0.000
Jitter ±4 s + ties (magnitude)	0.00	0.00	0.00	0.00	0.00

Under this tolerant rule, virtually all inversions induced purely by ±4 s jitter are treated as ties, and the simulated COI ratios collapse to zero. Empirical COI ratios in controls are already at this floor, while NT1 patients remain clearly above zero. Thus the qualitative conclusion that NT1 exhibits elevated COI relative to controls is robust to conservative assumptions about timing uncertainty and near-ties.

Table S17. Effect sizes and bootstrap confidence intervals for main group contrasts.

For each diagnostic group, this table reports patient–control contrasts for Z-AUC (primary endpoint), stage-occupancy entropy (bits), Euclidean deviation (ED), and the hemispheric Laterality Index. For each metric, it provides sample sizes, mean ± SD in patients and controls, Hedges' g with 95% bootstrap confidence intervals (5,000 resamples), Mann–Whitney p-values, and rank-biserial effect sizes. A separate panel B summarises the NT1 COI effect size, reporting mean ± SD COI in NT1 patients and controls along with Hedges' g, its bootstrap confidence interval, p-value and rank-biserial r. These effect sizes complement the main-text hypothesis tests by quantifying the magnitude and uncertainty of patient–control differences for primary and secondary microstructural metrics.

Panel A. Group contrasts for primary and secondary metrics.

Group	Metric	n_pat	n_ctrl	Patients mean ± SD	Controls mean ± SD	Hedges g	95% CI for g	p (Mann–Whitney)	Rank-biserial r
Fibromyalgia	Z-AUC	8	8	9.55 ± 7.61	0.00 ± 2.88	1.57	[0.76, 3.67]	0.015	-0.719
Fibromyalgia	Entropy (bits)	8	8	1.63 ± 0.50	1.92 ± 0.47	-0.57	[-1.77, 0.35]	0.279	0.344
Fibromyalgia	Euclidean deviation	8	8	8.87 ± 6.31	3.29 ± 0.96	1.17	[0.48, 2.57]	0.083	-0.531
Fibromyalgia	Laterality Index	8	8	-0.14 ± 0.27	-0.01 ± 0.04	-0.63	[-1.35, 0.67]	0.701	0.125
Narcolepsy	Z-AUC	12	12	-0.71 ± 5.35	-0.00 ± 2.51	-0.16	[-1.73, 0.51]	0.112	0.389
Narcolepsy	Entropy (bits)	12	12	1.84 ± 0.45	1.52 ± 0.75	0.50	[-0.28, 1.29]	0.371	-0.222
Narcolepsy	Euclidean deviation	12	12	4.03 ± 1.29	5.91 ± 3.87	-0.63	[-1.25, 0.06]	0.285	0.264
NREM Parasomnia	Z-AUC	16	16	-0.77 ± 2.13	0.00 ± 2.79	-0.30	[-0.99, 0.38]	0.585	0.117
NREM Parasomnia	Entropy (bits)	16	16	1.65 ± 0.65	1.65 ± 0.66	0.00	[-0.74, 0.65]	0.895	-0.031
NREM Parasomnia	Euclidean deviation	16	16	3.52 ± 1.35	4.67 ± 2.66	-0.54	[-1.17, 0.10]	0.250	0.242
NREM Parasomnia	Laterality Index	16	16	-0.07 ± 0.19	-0.03 ± 0.08	-0.28	[-0.81, 0.55]	0.724	-0.070

iRBD	Z-AUC	12	12	0.44 ± 3.55	0.00 ± 2.33	0.14	[-0.70, 0.97]	0.840	-0.056
iRBD	Entropy (bits)	12	12	1.67 ± 0.69	1.53 ± 0.64	0.20	[-0.56, 1.08]	0.707	-0.097
iRBD	Euclidean deviation (ED)	12	12	4.49 ± 2.07	6.06 ± 4.68	-0.42	[-1.00, 0.43]	0.583	0.139

As expected, the largest positive Hedges' g values are observed for Z-AUC in fibromyalgia (prolonged onset) and for COI in NT1 (see below), while effects for entropy, ED, and LI are more modest and often have confidence intervals that include zero, consistent with their exploratory status.

Panel B. NT1 COI effect size.

**Group**	**Metric**	**n**	**Mean ± SD**	**Hedges g**	**95% CI for g**	**p (Mann–Whitney)**	**Rank-biserial r**
Narcolepsy	COI	12	0.05 ± 0.07	0.92	[0.39, 1.55]	0.036	-0.333
Narcolepsy	COI controls	12	0.00 ± 0.00				

NT1 patients show an elevated COI ratio relative to matched controls (Hedges' g ≈ 0.92, 95% CI approximately [0.39, 1.55], p ≈ 0.036), with controls clustered at COI = 0. This supports the interpretation of NT1 as exhibiting irregular Hori ordering during sleep onset.

Panel C. NT1 entropy–TST correlation.

For the targeted microstructure–macrostructure pairing in NT1, we quantified the correlation between stage-occupancy entropy and total sleep time (TST) within the NT1 patient group and within its matched controls, and then examined the difference in correlation strength (Δr). Pearson correlations and 95% confidence intervals were estimated using non-parametric bootstrap resampling (5,000 iterations).

**Group**	**n**	**r (entropy–TST)**	**95% CI for r**	**p (two-sided)**	**Δr vs controls**	**95% CI for Δr**
Narcolepsy patients	12	0.345	[-0.18, 0.70]	0.272		
Narcolepsy controls	12	-0.380	[-0.76, 0.18]	0.223	0.725	[0.01, 1.26]

In this sample, entropy–TST coupling appears stronger in NT1 patients than in controls (Δr ≈ 0.72), and the bootstrap 95% CI for Δr narrowly excludes zero. However, both within-group CIs are wide, reflecting limited sample size, so these micro–macro associations are best viewed as preliminary and hypothesis-generating.

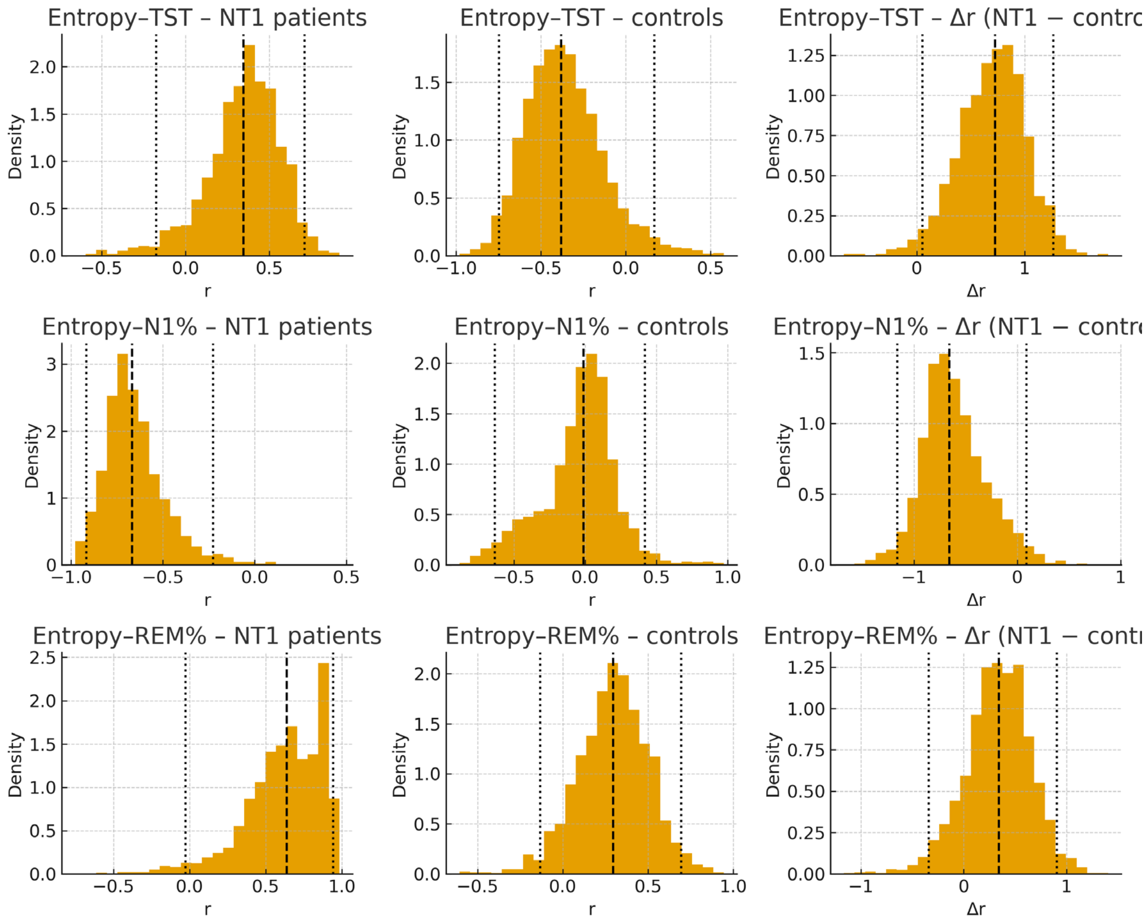

Figure S3. Bootstrap distributions for entropy–TST and related correlations in NT1. This figure displays bootstrap sampling distributions (5,000 resamples) for the Pearson correlation between stage-occupancy entropy and total sleep time (TST) in NT1 patients and controls, along with the distribution of Δr (r_patient − r_control). Additional panels show corresponding distributions for entropy–N1% and entropy–REM% as exploratory pairings. Vertical lines mark the empirical r values and the 95% bootstrap confidence intervals. The entropy–TST pairing shows a modest positive r in patients and a negative r in controls, with a Δr distribution whose 95% interval just excludes zero; the other pairings show qualitatively similar but less certain effects.

STROBE Checklist – Cross-sectional Observational Study

Manuscript title: Disordered Descent into Sleep: Microstructural Divergence Across Arousal-Linked Conditions

Study design: Retrospective exploratory cross-sectional clinical study with age- and sex-matched controls.

Setting: Sleep Disorders Centre, Guy's and St Thomas' NHS Foundation Trust (King's Health Partners/King's College London), London, UK; and Montreal Archive of Sleep Studies (MASS), Montreal, Canada.

Journal: npj Biological Timing and Sleep

STROBE item	Recommendation	Location in manuscript (section/paragraph)
Title and abstract	Indicate the study design in the title or abstract; provide an informative and balanced summary of what was done and found.	Title; unstructured Abstract (brief summary of Hori microstaging in four clinical groups and matched controls).
Background/rationale	Explain the scientific background and rationale for the investigation.	Opening 4–5 paragraphs of main text (from "The transition from wakefulness to sleep…" to "These theoretical frameworks are used here as an interpretive scaffold…").
Objectives	State specific objectives and any prespecified hypotheses.	Paragraph beginning "The present pilot study applies Hori microstaging…" (end of main-text introduction).
Study design	Describe the key elements of the study design early in the paper.	Methods – Participants (first two paragraphs).
Setting	Describe the setting, locations, and relevant dates, including periods of recruitment and data collection.	Methods – Participants; Methods – Multisite acquisition harmonisation and preprocessing; Supplementary Table S10 (acquisition parameters).
Participants	Give the eligibility criteria, and the sources and methods of selection of participants; describe methods of follow-up (if any).	Methods – Participants (inclusion/exclusion criteria, diagnostic definitions, matching); Supplementary Tables S1 and S11.

Variables	Clearly define all outcomes, exposures, predictors, potential confounders, and effect modifiers.	Methods – Hori Stage Scoring and Data Derivation; Laterality Index Calculation; Entropy and Trajectory Deviation Metrics; Cumulative Ordering Index; Stage-wise Z-Normalisation; Correlation Analysis; Statistical Analysis.
Data sources/measurement	For each variable of interest, give sources of data and details of methods of assessment; describe comparability of assessment methods in more than one group.	Methods – Hori Stage Scoring and Data Derivation; Multisite acquisition harmonisation and preprocessing; Supplementary Table S10.
Bias	Describe any efforts to address potential sources of bias (e.g. blinding, harmonisation, sensitivity analyses).	Methods – Hori Stage Scoring and Data Derivation (blinding, inter-rater reliability); Methods – Multisite acquisition harmonisation and preprocessing; Results – Cumulative ordering index (within-site analyses, null simulations); Results – Participant characteristics (AHI sensitivity); Supplementary Tables S10–S13, S15–S16; Supplementary Figures S1–S3.
Study size	Explain how the study size was arrived at.	Methods – Participants (retrospective identification of 48 eligible patients with complete Hori-scorable onset segments and 48 matched controls).
Quantitative variables	Explain how quantitative variables were handled in the analyses; describe groupings and transformations.	Methods – Stage-wise Z-Normalisation; Entropy and Trajectory Deviation Metrics; Cumulative Ordering Index; Correlation Analysis; Statistical Analysis.
Statistical methods	Describe all statistical methods, including those used to control for	Methods – Statistical Analysis; Correlation Analysis (Δr and bootstrap);

	confounding and those used to examine subgroups and interactions.	Visualisation; description of prespecified endpoints and exploratory analyses.
Participants (results)	Report numbers of individuals at each stage of study (e.g. eligible, included).	Results – Participant characteristics (numbers per diagnostic group and controls); Supplementary Table S1 (sample sizes).
Descriptive data	Give characteristics of study participants and information on exposures and potential confounders.	Results – Participant characteristics; Supplementary Table S1 (demographics, macrostructure); Supplementary Tables S11–S13 (age matching and AHI).
Outcome data	Report numbers of outcome events or summary measures.	Results – Microstructural sleep-onset profiles; Hemispheric asymmetry; Cumulative ordering index; Entropy and Euclidean distance; Stage-wise sleep-onset deviations; Microstructure–macrostructure correlations; Figures 1–3; Table 1; Supplementary Tables S2–S7 and S14–S17.
Main results	Give unadjusted estimates and, if applicable, confounder-adjusted estimates; make clear which confounders were adjusted for and why.	Results – main text (Z-AUC and NT1 COI as prespecified endpoints); Table 1; Supplementary Tables S1–S7 and S11–S17; Methods – Correlation Analysis (partial correlations).
Other analyses	Report other analyses done (e.g. subgroup analyses, sensitivity analyses).	Results – Cumulative ordering index (null simulations, tolerant ties); Hemispheric asymmetry (FM outlier removal); Microstructure–macrostructure correlations (partial correlations); Supplementary Tables S2, S7, S12–S17; Supplementary Figures S2–S3.

Key results	Summarise key results with reference to study objectives.	First three paragraphs of Discussion (summary of NT1 vs fibromyalgia patterns, main Z-AUC and COI findings).
Limitations	Discuss limitations of the study, taking into account sources of potential bias or imprecision.	Late Discussion paragraphs ("Several methodological considerations temper these inferences…").
Interpretation	Give a cautious overall interpretation of results considering objectives, limitations, multiplicity of analyses, and other relevant evidence.	Middle Discussion (sections on attractor framework, trajectories, psychiatric links), emphasising exploratory nature of several findings.
Generalisability	Discuss the generalisability (external validity) of the study results.	Final Discussion paragraphs, including comments on sample size, sex balance in fibromyalgia, multicentre design and need for replication.
